# New-generation rice seed germination assessment: high efficiency and flexibility via SeedRuler web-based platform

**DOI:** 10.3389/fpls.2025.1671998

**Published:** 2025-10-13

**Authors:** Zeyu Hou, Jinfeng Zhao, Sheng Dai, Jiawen Yang, Yan Ma, Ming Gong

**Affiliations:** ^1^ College of Information, Mechanical and Electrical Engineering, Shanghai Normal University, Shanghai, China; ^2^ Shanghai Key Laboratory of Plant Molecular Sciences, College of Life Sciences, Shanghai Normal University, Shanghai, China; ^3^ School of Education, Shanghai Normal University, Shanghai, China

**Keywords:** germination rate, germination standard, deep learning, image processing, object detection

## Abstract

**Introduction:**

The germination rate of rice seed is a critical indicator in agricultural research and production, directly influencing crop yield and quality. Traditional assessment methods based on manual visual inspection are often time-consuming, labor-intensive, and prone to subjectivity. Existing automated approaches, while helpful, typically suffer from limitations such as rigid germination standards, strict imaging requirements, and difficulties in handling the small size, dense arrangement, and variable radicle lengths of rice seeds.

**Methods:**

To address these challenges, we present SeedRuler, a versatile, web-based application designed to improve the accuracy, efficiency, and usability of rice seed germination analysis. SeedRuler integrates three core components: SeedRuler-IP, a traditional image processing-based module; SeedRuler-YOLO, a deep learning model built on YOLOv5 for high-precision object detection; and SeedRuler-SAM, which leverages the Segment Anything Model (SAM) for fine-grained seed segmentation. A dataset of 1,200 rice seed images was collected and manually annotated to train and evaluate the system. An interactive module enables users to flexibly define germination standards based on specific experimental needs.

**Results:**

SeedRuler-YOLO achieved a mean average precision (mAP) of 0.955 and a mean absolute error (MAE) of 0.110, demonstrating strong detection accuracy. Both SeedRuler-IP and SeedRuler-SAM support interactive germination standard customization, enhancing adaptability across diverse use cases. In addition, SeedRuler incorporates an automated seed size measurement function developed in our prior work, enabling efficient extraction of seed length and width from each image. The entire analysis pipeline is optimized for speed, delivering germination results in under 30 seconds per image.

**Conclusions:**

SeedRuler overcomes key limitations of existing methods by combining classical image processing with advanced deep learning models, offering accurate, scalable, and user-friendly germination analysis. Its flexible standard-setting and automated measurement features further enhance usability for both researchers and agricultural practitioners. SeedRuler represents a significant advancement in rice seed phenotyping, supporting more informed decision-making in seed selection, breeding, and crop management.

## 1 Introduction

The seed plays an indispensable role in agricultural production as a primary source of sustenance for humans (Zhao et al., 2023b; Rajalakshmi et al., 2024). The germination rate of seeds is an important factor in evaluating their quality and performance (Genze et al., 2020). Accurate and efficient assessment of germination rate is crucial for both crop genetic studies and breeding. Crop breeders utilize germination rate data to determine the optimal environment for seed germination, which greatly impacts seed storage and agriculture production (Colmer et al., 2020). In addition, with the accurate phenotyping of the germination rate of a genetic population, researchers can identify causal genes responsible for seed germination rate, thereby accelerating the breeding process (Pouvreau et al., 2013; Khoenkaw, 2016; Zhou et al., 2017).

In seed testing, the germination rate represents the percentage of seeds with protruding radicles among the total number of seeds tested under appropriate conditions and over a specified period of time (Urena et al., 2001; Chai et al., 2018). Typically, the germination rate is manually recorded by an experienced technician upon visual inspection of the Petri dishes for germinated seeds. However, this manual counting the number of germinating seeds is a time-consuming and error-prone task due to the small size of the seeds and the minimal color contrast between the seed coat and the radicle (Braguy et al., 2021), thereby limiting the frequency, scale, and accuracy of experiments.

In recent years, the field of germination tests has witnessed the application of computer vision and machine learning techniques. An example is the use of the k-nearest neighbors to analyze images of diverse germination phenotypes (Awty-Carroll et al., 2018). Joosen et al. (2010) developed a software package GERMINATOR (Joosen et al., 2010) that employs ImageJ, an open-source program, to determine Arabidopsis germination rates. Additionally, a computer vision germination system has been developed to determine the different categories of seeds during imbibition and germination (ElMasry et al., 2019). Furthermore, researchers performed high-throughput seed germination screening using 3D printed hole arrays along with image analysis software Image Pro Plus (Chai et al., 2018). Also, some researchers used threshold and maximum likelihood methods to evaluate the germination of pepper seeds (Chaivivatrakul, 2020). However, it is important to highlight that conventional image analysis methods may not be well-suited for conducting large-scale germination tests. This is primarily because manual parameter adjustments, such as threshold, can greatly affect their performance.

Unlike traditional image analysis methods, deep learning is capable of learning and extracting features and semantic information from images to identify, classify, and locate objects more effectively (Wei et al., 2024; Yao et al., 2024; Sangjan et al., 2025). For example, SeedQuant (Braguy et al., 2021) measures stimulant and inhibitor activity on root parasitic seeds using deep learning. Deep learning has been employed by researchers to predict germination rates for various plants, including tomatoes, peppers, barley, corn, and parasitic plants (Dheeraj and Chand, 2025; Maleki et al., 2025; Rezaei et al., 2025; Waseem et al., 2025). It should be noted, however, that the methods mentioned above all necessitate an interval between seeds, which is not convenient for practical purposes. YOLO-r (Zhao et al., 2023a) utilizes a convolutional neural network to assess the germination status of rice seeds, even when they are in contact with each other. However, a limitation is that users cannot manually customize the germination standards.

To address these issues, we developed a web-based platform called SeedRuler, which incorporates three methods: SeedRuler-YOLO, SeedRuler-SAM, and SeedRuler-IP. SeedRuler-YOLO is based on the object detection algorithm YOLOv5 (Redmon et al., 2016), which has demonstrated high accuracy and speed in various applications, including remote sensing detection (Adli et al., 2025), fruit detection (Tang et al., 2025), and pest detection (Wang et al., 2025). SeedRuler-SAM utilizes the Segment Anything Model (SAM) (Kirillov et al., 2023) for seed and radicle segmentation and calculates the ratio of their areas. SeedRuler-IP is based on image processing techniques. Both SeedRuler-SAM and SeedRuler-IP provide interactive functionality that enhances user flexibility and adaptability in selecting a germination standard. SeedRuler consists of the following functions: acquiring rice seed images, accurately positioning the seeds within the images, determining the germination status of each seed, and generating outputs of seed numbers and germination rates for all the captured images. Notably, SeedRuler allows rice seeds to be in contact with each other during image capture, enabling users to swiftly capture images and analyze rice seed germination with convenience.

The decision to implement SeedRuler as a web-based platform was driven by several practical considerations. First, a browser-accessible system removes the need for users to install additional software or maintain specific computing environments, which is especially useful for agricultural researchers with limited technical expertise. Second, the web-based architecture allows for centralized updates, consistent user experiences, and easier integration with cloud-based storage and processing resources. Compared with standalone desktop applications or mobile apps, a web platform enables better scalability, supports high-throughput batch processing, and allows cross-platform compatibility. Moreover, for users without stable internet access, we also provide an offline version with the same functionalities to ensure wide applicability.

Unlike existing tools, SeedRuler supports both fixed and customizable germination standards through a hybrid framework that integrates deep learning and interactive segmentation, offering greater adaptability across varied seed conditions. Each module in SeedRuler is designed to serve distinct application scenarios: SeedRuler-IP is suitable for low-computing-power environments or situations where internet access is limited; SeedRuler-YOLO offers high-speed and high-accuracy detection ideal for routine large-scale analysis; and SeedRuler-SAM supports fine-grained customization of germination standards, making it especially useful for detailed research or biological studies with specific phenotyping needs.

Despite these advantages, we acknowledge that SeedRuler, in its current version, does not offer fine-grained control over advanced algorithmic parameters. For instance, users cannot adjust low-level YOLOv5 model settings, segment-specific thresholds, or fine-tune detection sensitivity beyond the default interface options. This design prioritizes ease of use and accessibility over expert-level customization. Furthermore, while the image acquisition box ensures consistent image quality, the system’s performance may degrade when analyzing images captured under uncontrolled lighting or complex backgrounds. These limitations are discussed further in the Discussion section, and we plan to address them in future updates.

In conclusion, SeedRuler can perform large-scale rice seed germination phenotyping and holds potential applications in seed genetic studies and agricultural production due to its high accuracy, speed, affordability, and user-friendly interface. SeedRuler is available for free (http://www.xhhuanglab.cn/tool/SeedRuler.html) and is compatible with Windows, MacOS, and Linux operating systems.

## 2 Materials and methods

### 2.1 Materials

The rice sources used in this study were obtained from *indica* and *japonica* varieties in the field conducted by Shanghai Normal University (Xuehui Huang Lab). This population of rice seeds exhibits a broad range of phenotypic variation among different lines.

In the germination experiment, 30–60 full-grained seeds were randomly selected from each rice cob and transferred to Petri dishes. Next, add the appropriate amount of distilled water to each Petri dish (to prevent water evaporation, seal the Petri dish with plastic wrap). Subsequently, the petri dish was positioned in a 25°C constant temperature incubator for dark culture. Finally, seed images were obtained by photographing germinating seeds. In this study, all seeds were acquired through the image acquisition box between October 2020 and September 2021.

### 2.2 Data set generation

To reduce interference from factors such as camera lens distortion and external lighting, we have specially designed an image acquisition box. The acquisition box is equipped with an internal light source and the focal length of the camera is fixed, which ensures excellent image quality.

The image acquisition box consists of three main components: the camera, the box body, and the light source. The camera has a focal length of 2.8-12mm and a resolution of 1920×1080. The rectangular box body has dimensions of 30cm×30cm×15cm. The inner wall of the box body is made of highly reflective granular fabric, which allows the light to be diffused evenly. Circular LED lights beneath the image acquisition port serve as the light source. A ring-shaped LED light source is located on top of the box. The light source casts its illumination onto the top of the box, which is then reflected by both the top and side walls, resulting in the generation of diffuse light at the bottom of the box.

In the imaging process, the seed-containing Petri dish is placed under the camera in the image acquisition box and photographed, resulting in a 1920×1080 image which is stored in the computer as a JPEG file. It takes at most half a minute to capture one seed image using the image acquisition box. A technician can capture 1,000 images in a single day if he shoots continuously for eight hours. Hence, the use of an image acquisition box (**Additional File 1**: **Supplementary Figure S1**) can greatly improve the efficiency of the shooting process.

To ensure the diversity of the dataset, we collected 1200 seed images with a total of 4,4660 seeds. Each image contains approximately 30–60 seeds. The images contain seeds of varying size, shape, and color, along with impurities like branch stalks, fragmented leaves, and rice awns. Additionally, the distribution of seeds in the image is sparsely distributed.

After acquiring the images, the technician used LabelImg to label each seed with a rectangular box and define its category. Here, the seed categories include germinated and ungerminated seeds that are labeled with “yes” and “no”, respectively, and the labeling results are shown in **Additional File 1**: **Supplementary Figure S2**. The dataset is then randomly split into training and test sets, ensuring an 8:2 ratio. The training and test sets consist of 960 and 240 images, respectively, for training and testing the model.

### 2.3 Algorithm

#### 2.3.1 SeedRuler-IP

**Figure 1:**
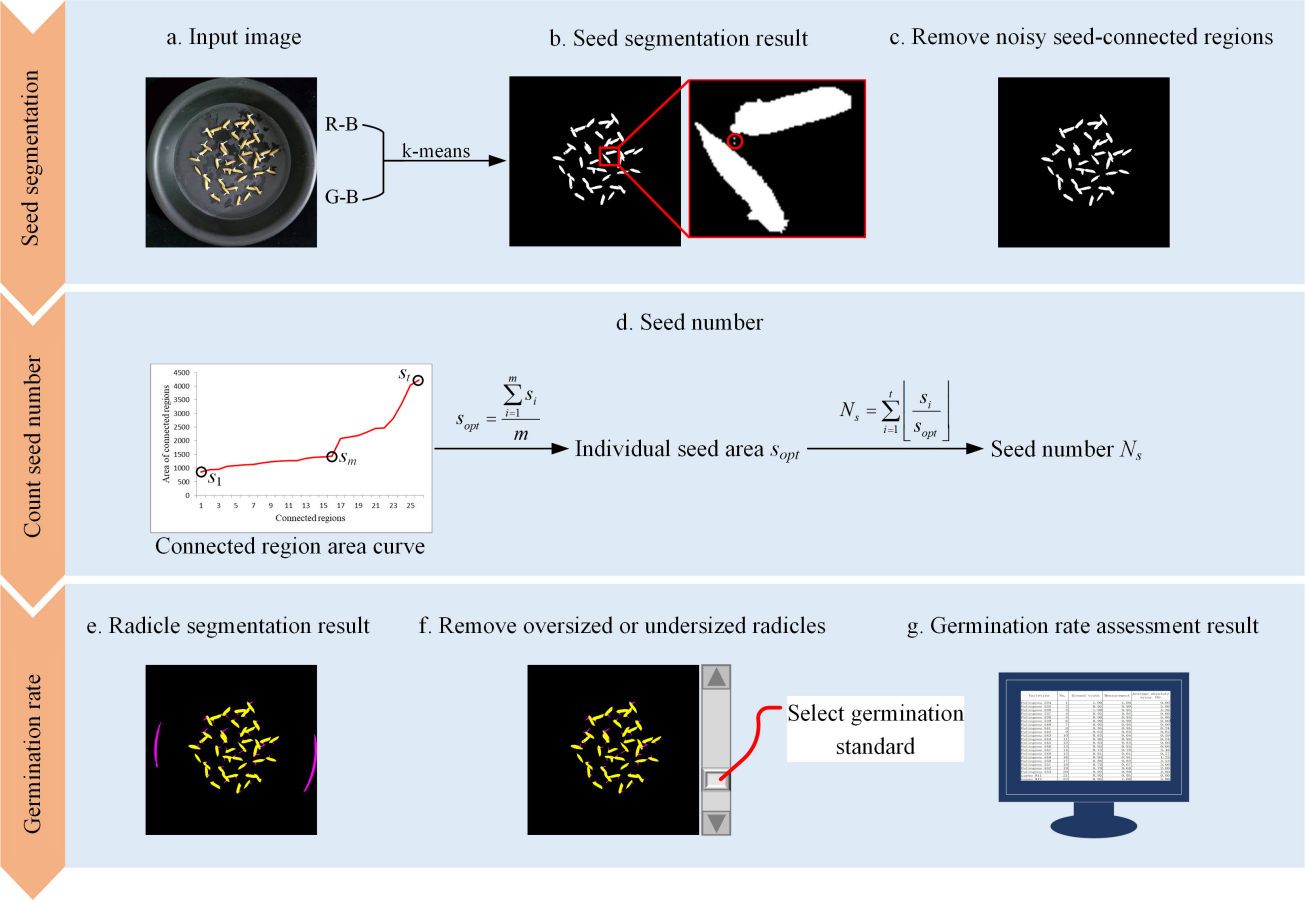
Flow chart for SeedRuler-IP. First, the k-means clustering algorithm is employed on the input image to separate seeds from the background **(A, B)**. Then, noisy seed-connected regions are eliminated **(C)**. Subsequently, the area curve of the connected regions is utilized to determine the individual seed area and the number of seeds **(D)**. Next, pixels with R, G, and B values exceeding a threshold are classified as radicles, while excessively large or small radicles are removed. Finally **(E, F)**, the user interacts by adjusting a scrollbar to determine the radicle length standard, resulting in the germination rate of the input image **(G)**.

SeedRuler-IP assesses seed germination rate using a classical image processing pipeline, consisting of color-based segmentation, morphological refinement, seed counting, and radicle identification. The workflow is illustrated in **Figure 1**. First, k-means clustering is applied to the input image to separate seeds from the background (a, b), followed by the removal of noisy connected regions (c). The area curve of the connected regions is then used to determine the number and size of seeds (d). Pixels exceeding preset RGB thresholds are classified as radicles, and abnormally large or small radicles are filtered out (e, f). Finally, the user adjusts a scrollbar to set the radicle length threshold, and the system calculates the germination rate accordingly (g). Below are the detailed steps.

##### 2.3.1.1 Preprocessing and seed segmentation

To reduce noise and enhance edge information, a Gaussian filter with a kernel size of 3×3 is first applied to the input image. Then, each pixel’s RGB values (*R*, *G*, *B*) are transformed into two new features:


(1) $$f_{1}=R-B,\;f_{2}=G-B$$

This transformation enhances the contrast between seed coat pixels (typically yellowish, with high *R* and *G*) and background or radicle pixels (typically dark or white, with balanced RGB). These two features are used as input for K-means clustering with the following settings:

i. Number of clusters: *k*=2.
ii. Initial cluster centers: (0, 0) and (30, 30).
iii. Stopping criterion: clustering terminates when cluster centers converge.

Let the resulting two clusters be *C*_1_ and *C*_2_, where *C*_1_ (higher feature values) corresponds to seed coat pixels, and *C*_2_ (lower feature values) corresponds to background and radicle.

##### 2.3.1.2 Morphological refinement and seed region extraction

Seed pixels from *C*_1_ are grouped into connected components. Morphological operations are applied to remove small noise regions with area<200 pixels. Let the set of remaining connected components be ${\left\{R_{i}\right\}}_{i=1}^{N}$ , with area $\left\{s_{i}\right\}$ . These areas are sorted in ascending order. To estimate the average single-seed area *s_opt_*, a difference-based stability check is performed. Starting from the second component, we compute:


(2) $${\text{Δ}}_{i}=s_{i+1}-s_{i}$$

The region is considered stable when ${\text{Δ}}_{i}<30$ for at least two consecutive components. All components before the first unstable region are used to compute:


(3) $$s_{opt}=\frac{1}{m}\sum_{i=1}^{m}s_{i}$$

where *m* is the number of stable components. The total number of seeds is estimated as:


(4) $$N_{s}=\sum_{i=1}^{t}\lfloor \frac{s_{i}}{s_{opt}}\rfloor$$

##### 2.3.1.3 Radicle detection and germination assessment

Pixels in cluster *C*_2_ with *R*>160, *G*>160, and *B*>160 are classified as radicle candidates. These pixels are further grouped into connected regions, and morphological filtering is applied to remove extremely small or large areas.

To assess germination, SeedRuler-IP allows the user to interactively set a germination threshold *Tg* using a graphical scrollbar. For example, if *T_g_* =100, all radicle regions with area>100 are considered as germinated. The final germination rate is calculated as:


(5) $$\text{Germination rate=}\frac{N_{r}}{N_{s}}$$

where *N_r_* represents the number of germinated seeds.

#### 2.3.2 SeedRuler-YOLO

In recent years, significant progress has been achieved in object detection research (Viola and Jones, 2001; Su et al., 2017). The YOLO model (Redmon and Farhadi, 2017) is capable of localizing and categorizing objects of different sizes, i.e., marking them with a minimum external rectangle and assigning them a category. In this study, we developed high-precision and high-efficiency software for evaluating rice seed germination rate based on the YOLOv5 model.

YOLOv5 has three parts: input, backbone, and head (**Additional File 1**: **Supplementary Figure S3**). The head includes the neck and detection. The input layer has a 640×640×3 image. We utilized four different versions of YOLOv5, namely YOLOv5s, YOLOv5m, YOLOv5l, and YOLOv5x. The four versions have similar network structures, with an increase in network depth and the number of convolutional kernels in proportion to the version, subsequently improving network performance, but at the cost of reduced running speed. The network structures of the four versions are detailed in **Additional File 1**: **Supplementary Table S1**. We trained and tested these four models to compare their seed germination detection results. It is necessary to balance the accuracy and speed of the model to achieve satisfactory results (Sindagi and Patel, 2018).

**Figure 2:**
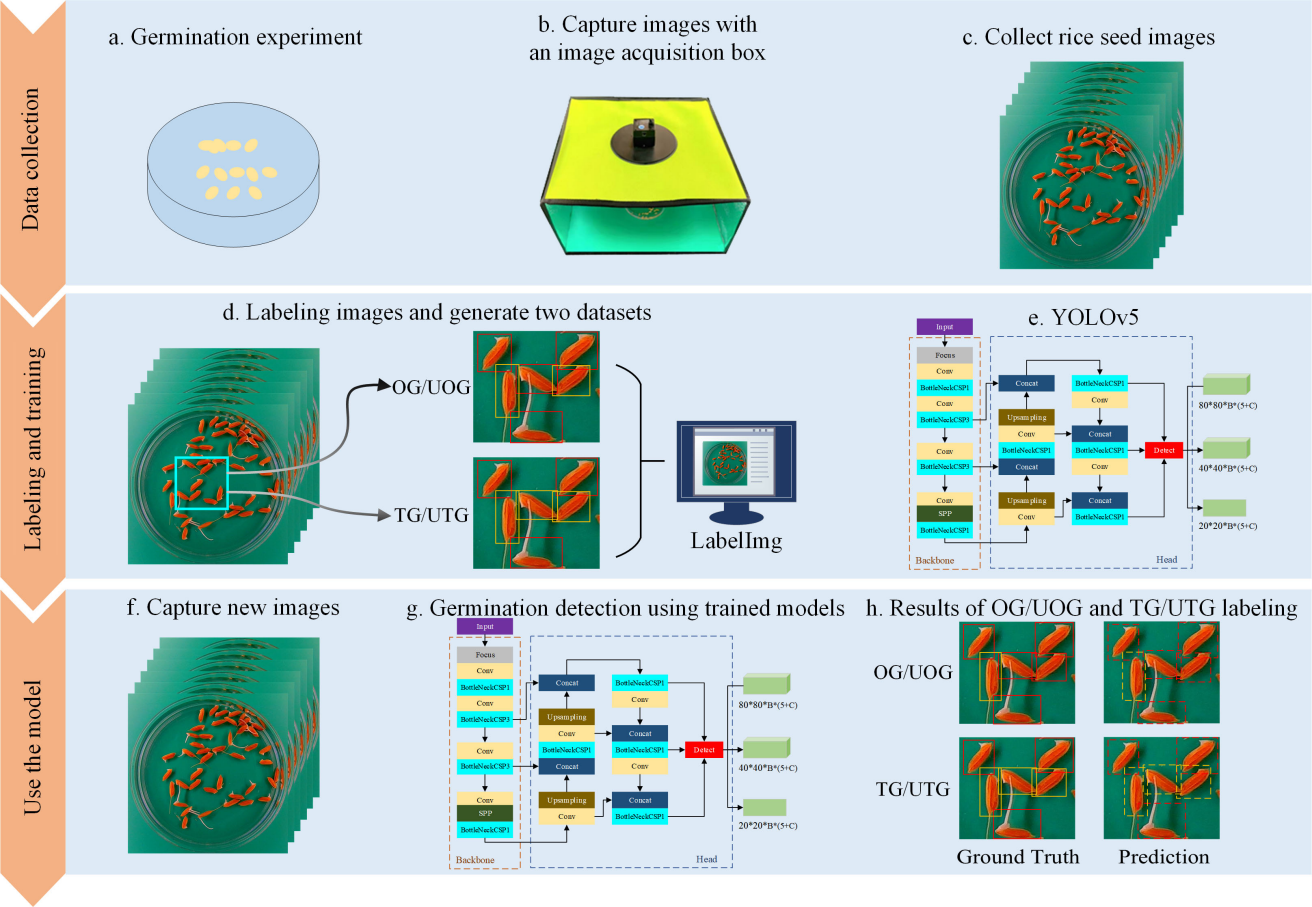
Flow chart for SeedRuler-YOLO. During the data collection phase, seeds to be germinated were placed in Petri dishes for germination experiments **(A)**. Meanwhile, we developed an image acquisition box specifically designed for capturing high-quality digital photographs **(B)**. Next, we placed the Petri dish containing the germinating rice seeds directly under the camera of the image acquisition box to take a picture **(C)**. Next, each germinated or ungerminated seed in the image was labeled by an experienced technician, i.e. the seeds were marked with a minimum external rectangle using LabelImg software **(D)**. Here, two germination standards, OG/UOG and TG/UTG, were used respectively. Afterward, we trained four network structures, YOLOv5s, YOLOv5m, YOLOv5l, and YOLOv5x **(E)**. Following the training of each of the four network models, new images were input to detect germinated seeds **(F, G)**. Finally, the model performance was evaluated by comparing the detection results with ground truth **(H)**.

As a first step, rice seeds were placed in Petri dishes for germination culture. The seed images were then acquired using an image acquisition box. Afterward, germinated and ungerminated seeds were labeled using LabelImg software, resulting in the creation of a seed image dataset. Finally, four YOLOv5 network structures were trained to obtain the corresponding automatic seed detection models. The experimental flow chart, depicted in **Figure 2**, illustrates the process. During the data collection stage, rice seeds were placed in Petri dishes for germination experiments (a), and an image acquisition box was specifically developed to capture high-quality digital photographs of the samples (b). The Petri dish was positioned directly under the camera to obtain clear top-view images (c). Each seed in the image was then annotated by an experienced technician using a minimum external rectangle via LabelImg software, with both OG/UOG and TG/UTG germination standards applied (d). Four YOLOv5 model variants—YOLOv5s, YOLOv5m, YOLOv5l, and YOLOv5x—were trained using the labeled datasets (e). After training, the models were used to detect germinated seeds in new images (f, g), and their performance was evaluated by comparing predictions against ground truth annotations (h).

To obtain an accurate rice seed detection model, we established two seed germination standards to label seeds for different radicle lengths: (1) OG/UOG. A germinated seed (OG, where ‘O’ represents one mm) has a radicle length greater than 1mm, while an ungerminated seed (UOG) does not. (2) TG/UTG. A germinated seed (TG, where ‘T’ represents two mm) has a radicle length greater than 2mm, while an ungerminated seed (UTG) does not. The radicle length of each seed was assessed by trained technicians to determine if it exceeded 1mm or 2mm. Our experiments indicated that the OG/UOG standard defined germination lengths that were too short, potentially leading to mislabeling. In the TG/UTG standard, the radicle length is defined as 2mm, which facilitates determining whether seeds are germinating and ensures accurate labeling. Following the annotation of all seed images, we obtained two seed image datasets corresponding to OG/UOG and TG/UTG germination standards, respectively. Then, four network models, YOLOv5s, YOLOv5m, YOLOv5l, and YOLOv5x, were trained.

To ensure reproducibility, the training process of the YOLOv5 models was configured as follows: the input image size was set to 640×640, batch size to 64, and the optimizer used was SGD. The initial learning rate was 0.01, and the training was set to run for a maximum of 300 epochs. An early stopping mechanism was applied, where training would terminate if the loss did not decrease for 50 consecutive epochs. No additional data augmentation techniques were applied beyond the default YOLOv5 pipeline.

#### 2.3.3 SeedRuler-SAM

To address the limitation of rigid germination criteria in traditional models, we introduce SeedRuler-SAM, a semi-automatic segmentation-based module that allows for interactive customization of germination standards based on radicle length. The method combines the detection capability of SeedRuler-YOLO with the segmentation precision of the Segment Anything Model (SAM), enabling fine-grained and flexible germination assessment.

The SeedRuler-SAM process consists of the following steps:

##### 2.3.3.1 Initial detection

SeedRuler-YOLO is first used to detect all visually germinated seeds in the input image. Each detected object is enclosed in a bounding box.

##### 2.3.3.2 Interactive standard setting

The user interactively selects one seed in the image as the reference seed, which serves as the germination benchmark. The SAM algorithm is then applied to segment this reference seed, separating the radicle and seed body.

##### 2.3.3.3 Segmentation and ratio calculation

For each seed within its bounding box, SAM is used again to perform segmentation. Given that the radicle is typically white in color, the segmented region is divided into two parts:

- Pixels with R, G, and B values greater than a threshold (e.g., 100) are considered radicle pixels.
- The remaining pixels are treated as seed body.

The area ratio *r* is computed as the area of the radicle divided by the total area of the segmented seed. Similarly, for the reference seed, a ratio *r*_0_ is calculated. If *r* ≥ *r*_0_, the seed is classified as germinated; otherwise, it is non-germinated.

##### 2.3.3.4 Output and reporting

The germination status of all seeds is compiled, and the final results are exported in Excel format, including the total number of germinated seeds and the germination rate.

**Figure 3:**
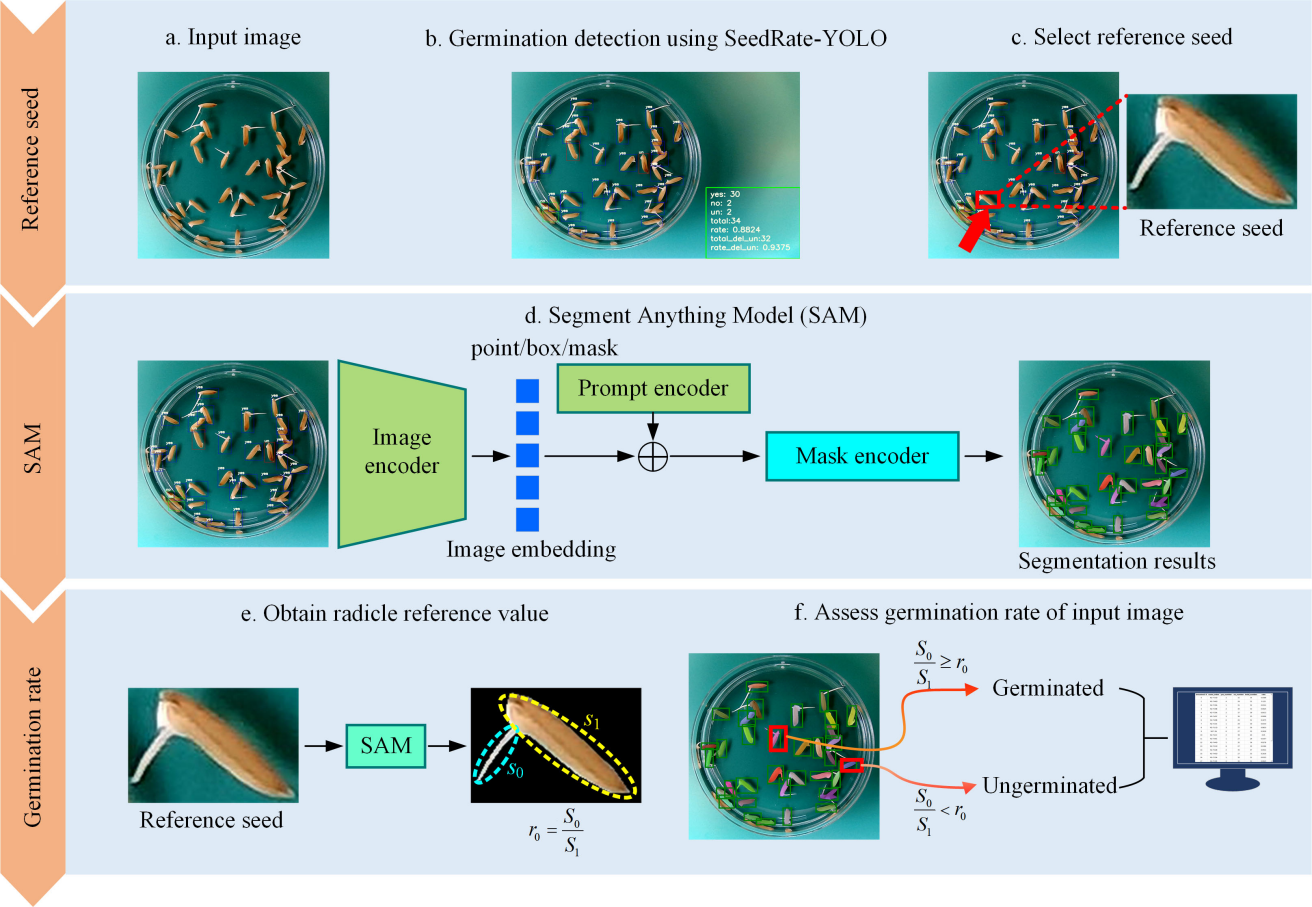
Experimental flow chart for SeedRuler-SAM. First, utilize SeedRuler-YOLO for germination detection in the input image **(A, B)**. Then, users interactively select the reference seed **(C)**. Next, use the Segment Anything Model (SAM) to segment the seeds and radicle within the bounding box **(D)**. Afterward, employ SAM to separate the reference seed from the background and calculate the ratio of sprout area to seed area, denoted as *r*_0_ **(E)**. Finally, evaluate the germination rate of the input image using radicle reference values and output the germination rates for each image in an Excel file format **(F)**.

**Figure 3** illustrates the full experimental flow of SeedRuler-SAM. After YOLO-based detection (a, b), the user selects a reference seed (c). SAM is then applied to segment all seeds and radicles (d), and the germination standard is computed from the reference seed (e). Finally, the germination status is determined and reported (f).

### 2.4 Implementation and functional modules of the SeedRuler web server

SeedRuler is a comprehensive rice seed germination analysis platform that integrates both an online web server and an offline software package. It is designed to provide high-performance, user-friendly, and flexible germination detection capabilities for agricultural research and seed phenotyping.

#### 2.4.1 System architecture

The web server is developed using the Layui framework for the user interface, in combination with Bootstrap to enhance frontend responsiveness. On the backend, the system is developed using Spring, Spring MVC, and MyBatis, ensuring robust and efficient data processing. MySQL is used as the database management system to ensure reliable and concurrent access to stored data. The server operates on Tomcat and is deployed on a Linux operating system equipped with Intel Xeon E5–2680 v4 processors and RTX 3060 GPUs, allowing for high-throughput processing and real-time response to user requests.

In addition to the web server, we provide an offline software package that supports the same functionalities and is built using PyQt5 for cross-platform GUI development. All algorithms and models are implemented in Python 3.8, with dependencies including PyTorch, NumPy, Pillow, and others. The offline software is compatible with Windows, macOS, and Linux. Users can download the package, along with a comprehensive user manual, from http://www.xhhuanglab.cn/tool/SeedRuler.html. This manual includes detailed operational procedures for using each module, instructions for environment setup (including CUDA 10.1 support), and guidance for batch processing, seed selection, and result export. These resources are intended to ensure reproducibility and facilitate adoption by other researchers.

#### 2.4.2 Functional modules and workflow

As illustrated in **Figure 5**, the SeedRuler platform supports a complete workflow for rice seed germination evaluation. The process begins with the selection of plump rice seeds, which are placed in Petri dishes for germination experiments. An image acquisition box is then used to capture high-quality images of the Petri dishes (**Figure 5A**).

**Figure 4:**
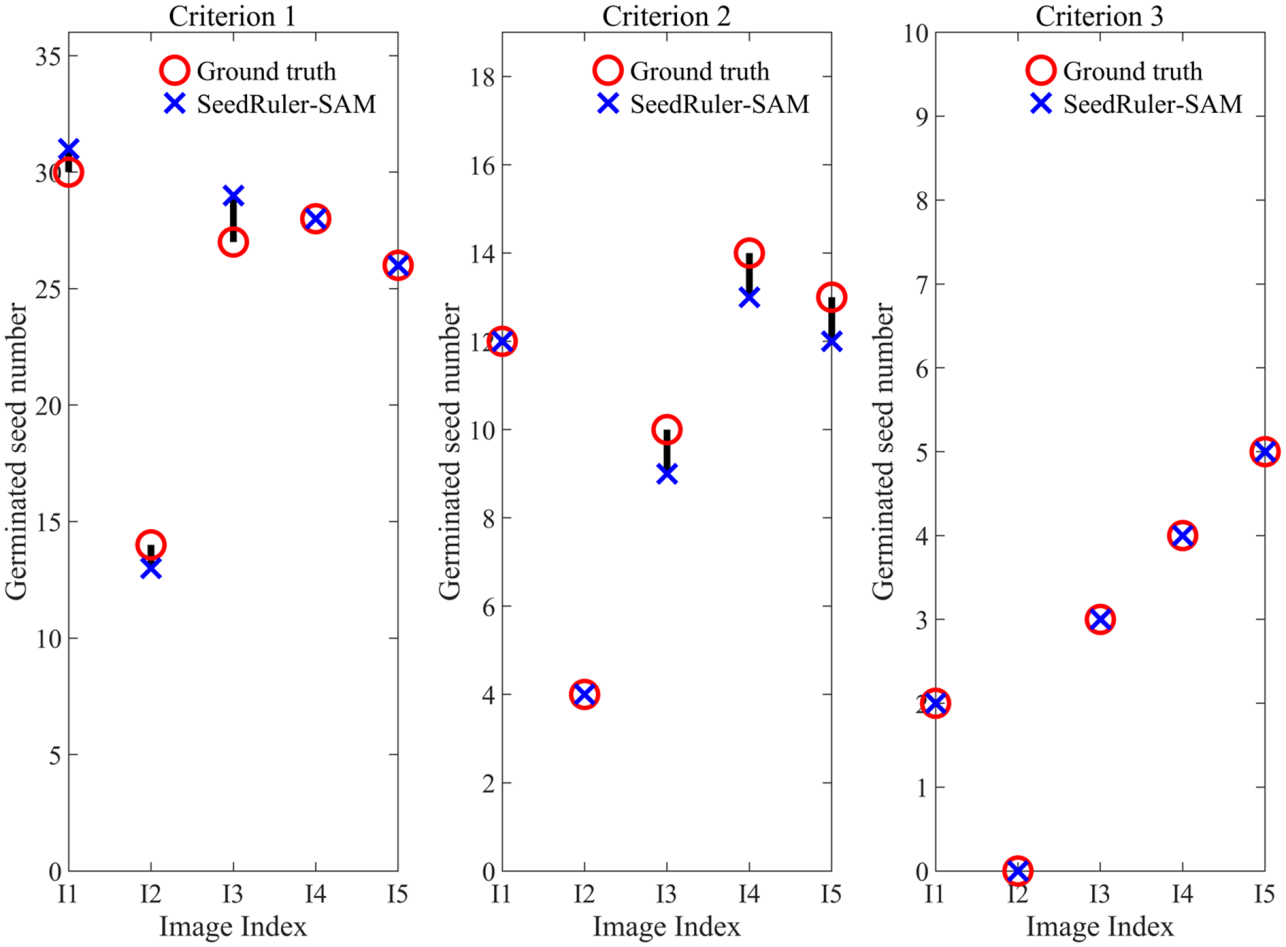
Estimate the germinated seed number of five images (I1, I2, I3, I4, I5) using SeedRuler-SAM under three germination standards.

**Figure 5:**
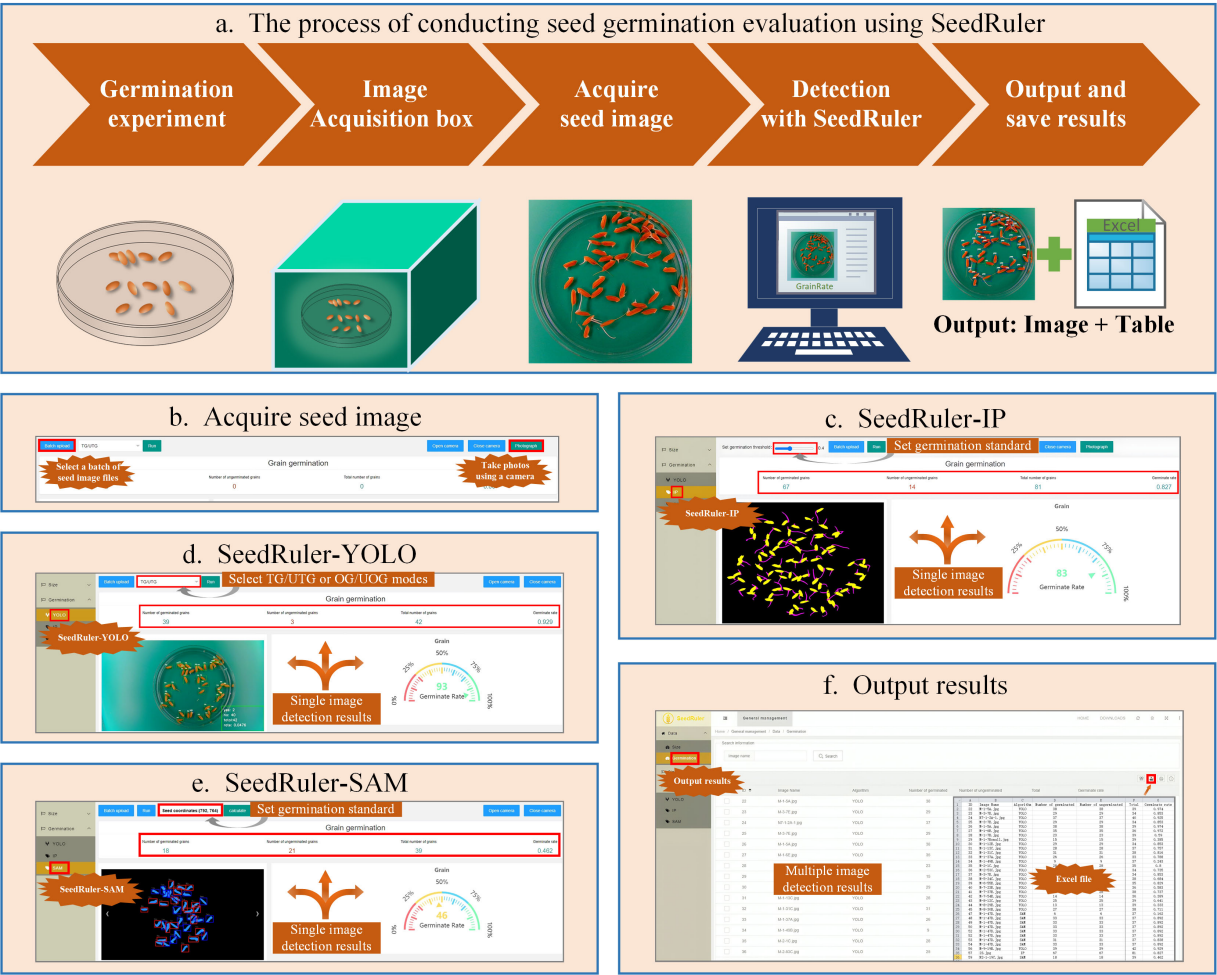
Functional Modules of the SeedRuler Web Server. Rice seed germination evaluation with SeedRuler involves selecting full seeds, placing them in Petri dishes, and capturing images with an image acquisition box **(A)**. SeedRuler offers two methods for acquiring seed images: “Batch Upload” and “Photograph” **(B)**. Three detection algorithms, SeedRuler-IP, SeedRuler-YOLO, and SeedRuler-SAM, analyze the images to generate detection results and germination rates **(C–E)**. The user-friendly interface allows graphical manipulation and quick export of detection results to tables, including seed name, germination counts, and rates **(F)**.

Seed images can be acquired in one of two ways: “Batch Upload”, which allows users to upload multiple images at once, or “Photograph”, which uses a computer’s built-in or external camera to capture images directly (**Figure 5B**). Once images are uploaded, users can choose from three available germination detection algorithms:

- SeedRuler-IP (**Figure 5C**): Based on traditional image processing, this module allows users to adjust the germination threshold using a graphical slider.
- SeedRuler-YOLO (**Figure 5D**): A deep learning-based module utilizing YOLOv5, which offers TG/UTG and OG/UOG detection modes.
- SeedRuler-SAM (**Figure 5E**): Built on the Segment Anything Model (SAM), this module enables users to interactively select a seed as a germination reference standard using mouse input.

All three modules generate annotated output images and calculate germination rates for each processed image. Users can view results by selecting the “Germination” option in the “Data” menu. Detection results can be exported individually or in batches to Excel (XLS) files, which include key information such as seed name, germinated seed count, total number of seeds, and germination rate (**Figure 5F**).

#### 2.4.3 Additional functionalities

To further support seed phenotyping tasks, SeedRuler includes an automated seed size measurement function (Zhao et al., 2023a), which enables rapid extraction of seed length and width from each image. This feature is seamlessly integrated into the detection pipeline and is particularly useful for combining germination rate analysis with morphological trait assessment.

## 3 Results

### 3.1 Evaluation indicators

Next, we need to run the completed training YOLOv5 model on the test image set for statistics and analysis. Here, we use mAP and MAE to evaluate the results **Equations 6**–**17** present the details of the algorithm.

The average precision (mAP) is a significant evaluation metric utilized to assess the performance of the model (Huang et al., 2020). Before proceeding with the evaluation, it is essential to provide clear definitions for true positive (TP), false positive (FP), and false negative (FN).

TP: It refers to the situation where the IoU value between the predicted bounding box and the ground truth bounding box is higher than the specified threshold.
FP: It denotes the scenario where the IoU value between the predicted bounding box and the ground truth bounding box is lower than the specified threshold.
FN: It refers to the situation where the ground truth exists, indicating the presence of an object, but the model fails to predict any bounding box for that object.

Based on the above definition, we can calculate the precision and recall:


(6) $$\text{Precision}=\frac{TP}{TP+FP}$$

(7) $$\text{Recall}=\frac{TP}{TP+FN}$$

And mAP is defined as follows:


(8) $$AP=\int_{0}^{1}P(R)dR$$

where *P* and *R* are Precision and Recall, respectively.


(9) $$mAP=\frac{AP}{k}$$

where *k* represents the number of classes.

In addition, we used MAE to evaluate the results:


(10) $$AE=\frac{1}{M}\sum_{i=1}^{M}\frac{abs(y_{i}-y_{i}^{'})}{y_{i}}$$

(11) $$mAE=\frac{1}{N}\sum_{i=1}^{N}AE_{i}$$

where *M* denotes the number of images, *y_i_* denotes the ground truth value for the *i*th image, denotes the prediction of the model for the *i*th image, *N* denotes the class number, and *AE_i_* denotes the *AE* value of the *i*th class object.

In addition to mAP and MAE, we also adopted four commonly used evaluation metrics to assess the segmentation performance of different models: Dice coefficient (Dice), Intersection over Union (IoU), Pixel Accuracy (PA), and False Positive Rate (FPR).

The Dice coefficient measures the overlap between the predicted segmentation and the ground truth. It is defined as:


(12) $$Dice=\frac{2\times TP}{2\times TP+FP+FN}$$

where *TP* denotes the number of true positive pixels, *FP* is the number of false positives, and *FN* is the number of false negatives.

The Intersection over Union (IoU), also known as the Jaccard index, evaluates the ratio between the intersection and the union of the predicted region and the ground truth region. It is given by:


(13) $$IoU=\frac{TP}{TP+FP+FN}$$

The Pixel Accuracy (PA) represents the proportion of correctly classified pixels over the total number of pixels in the image. It is calculated as:


(14) $$PA=\frac{TP+TN}{TP+TN+FP+FN}$$

where *TN* refers to the number of true negative pixels.

The False Positive Rate (FPR) indicates the proportion of background pixels that are incorrectly predicted as foreground. It is defined as:


(15) $$FPT=\frac{FP}{FP+TN}$$

### 3.2 Evaluation of SeedRuler-IP

**Figure 6:**
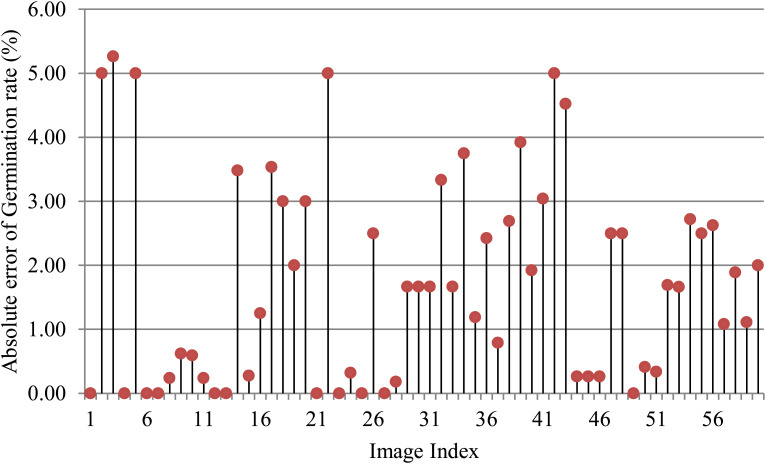
Absolute error of germination rate for 60 images.


To assess the effectiveness of SeedRuler-IP, we conducted experiments on 60 seed germination images. These images exhibited variations in terms of rice varieties, seed number, radicle length, and lighting conditions (**Additional File 1**: **Supplementary Figure S4**). **Figure 6** presents the absolute error of germination rates for the 60 images, where a germination standard of 0.4 was chosen for the analysis. It should be noted that the germination standard is defined as an area ratio between the radicle and its seed body. The specific formula for the area ratio is as follows:

(16) $$Area\;ratio=\frac{r}{s}$$

where *r* and *s* represent the area of the connected region belonging to the radicle or seed body, respectively. From **Figure 6**, it can be observed that except for five images with absolute errors greater than or equal to 5%, the absolute errors for the remaining images were below 4%, which demonstrates the superiority of SeedRuler in accurately estimating germination rate.

### 3.3 Evaluation of SeedRuler-YOLO

To evaluate the capability of YOLOv5 in identifying germinated or ungerminated seeds, we developed an image acquisition box to capture high-quality images. Also, we compared the model performance under different germination standards. Our experiments revealed that different germination standards do impact the performance of the model to some extent. We categorized the seed images using two standards, OG/UOG and TG/UTG, and obtained two datasets as a result. Consequently, we compared the performance of the models trained on these two datasets.

**Figure 7:**
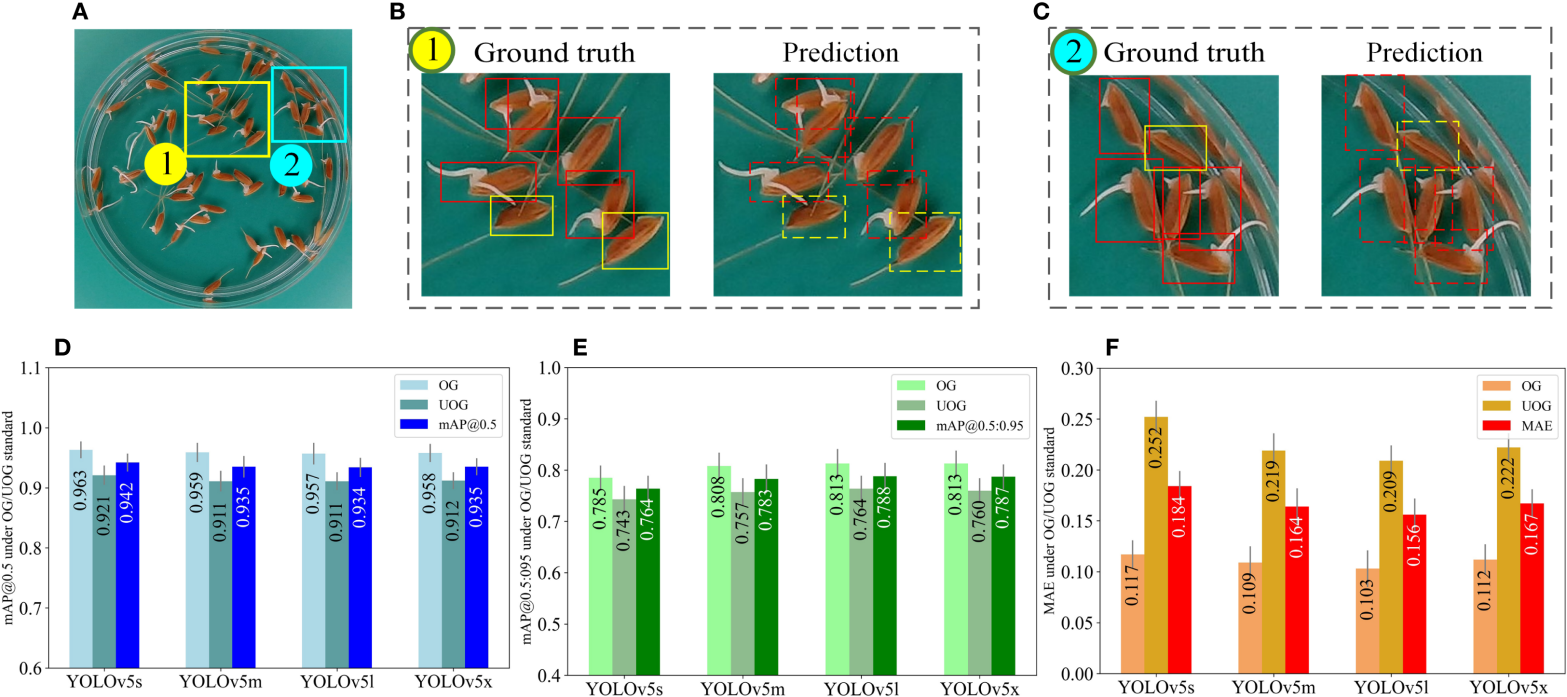
Model performance evaluation under the OG/UOG standard. **(A)** is a germinated seed image. The rectangular boxes in **(B, C)** indicate the ground truth (left) and the predicted results of YOLOv5m (right), where the red boxes indicate the germinated seeds and the green boxes indicate the ungerminated seeds. **(D–F)** represent the mAP@0.5, mAP@0.5:0.95, and MAE values obtained from testing 240 seed images using four YOLOv5 models (YOLOv5s, YOLOv5m, YOLOv5l, YOLOv5x), respectively (three decimal places are preserved), where error bars represent standard deviation.

**Figure 7** compares the detection performance of four YOLOv5 models under the OG/UOG germination standard. Panel (a) shows a representative image of germinated seeds, while (b) and (c) compare the ground truth annotations (left) with the YOLOv5m prediction results (right), where germinated seeds are marked with red boxes and ungerminated ones with green boxes. Panels (d), (e), and (f) present the evaluation metrics—mAP@0.5, mAP@0.5:0.95, and MAE—calculated from 240 test images using four YOLOv5 models (YOLOv5s, YOLOv5m, YOLOv5l, and YOLOv5x). The results are reported to three decimal places, and error bars indicate the standard deviation, reflecting the consistency and reliability of each model’s performance.

Initially, we exploited the image acquisition box to capture images. Next, an OG/UOG germination standard is defined, i.e. seeds with radicle lengths greater than 1mm are considered to have germinated, otherwise, they have not (**Figures 7A–C**). As demonstrated in **Figures 7D, E**, YOLOv5m exhibited a mAP@0.5 of 0.935 and a mAP0.5:0.95 of 0.783, while YOLOv5l showcased a mAP@0.5 of 0.934 and a mAP0.5:0.95 of 0.788. Furthermore, the average detection times of YOLOv5s, YOLOv5m, YOLOv5l, and YOLOv5x were recorded as 4 ms, 8.6 ms, 15 ms, and 30 ms per image, respectively. Additionally, in terms of MAE, the MAE values of YOLOv5m and YOLOv5l were lower than those of the other 2 models. Therefore, YOLOv5m and YOLOv5l are more suitable for germination experiments due to their accuracy and detection speed. **Figure 7F** illustrates that the MAE values of all four models are larger than 0.2 for ungerminated seeds. This outcome can be attributed to the 1mm germination standard being relatively short, leading to the misidentification of ungerminated seeds as germinated.

To further assess the agreement between predicted and ground-truth germination counts under the OG/UOG standard, we computed the relative error (RE) for each test image as follows:


(17) $$RE=\frac{\left|\text{Prediction}-\text{Ground Truth}\right|}{\text{Ground Truth}}\times 100\%$$

Across the 240 test images, the average relative error was 6.3% for germinated seeds and 8.7% for ungerminated seeds using the YOLOv5m model, which achieved the best trade-off between speed and accuracy. These results indicate that the prediction is generally close to the ground truth.

To confirm whether there is a statistically significant difference between predicted and true counts, we conducted a paired *t*-test comparing the predicted and manually annotated germination rates across all test images. The *p*-value obtained was 0.31 (*p* > 0.05), indicating no statistically significant difference between the two. This supports the conclusion that the model’s output is consistent with human annotation under the OG/UOG standard.

**Figure 8:**
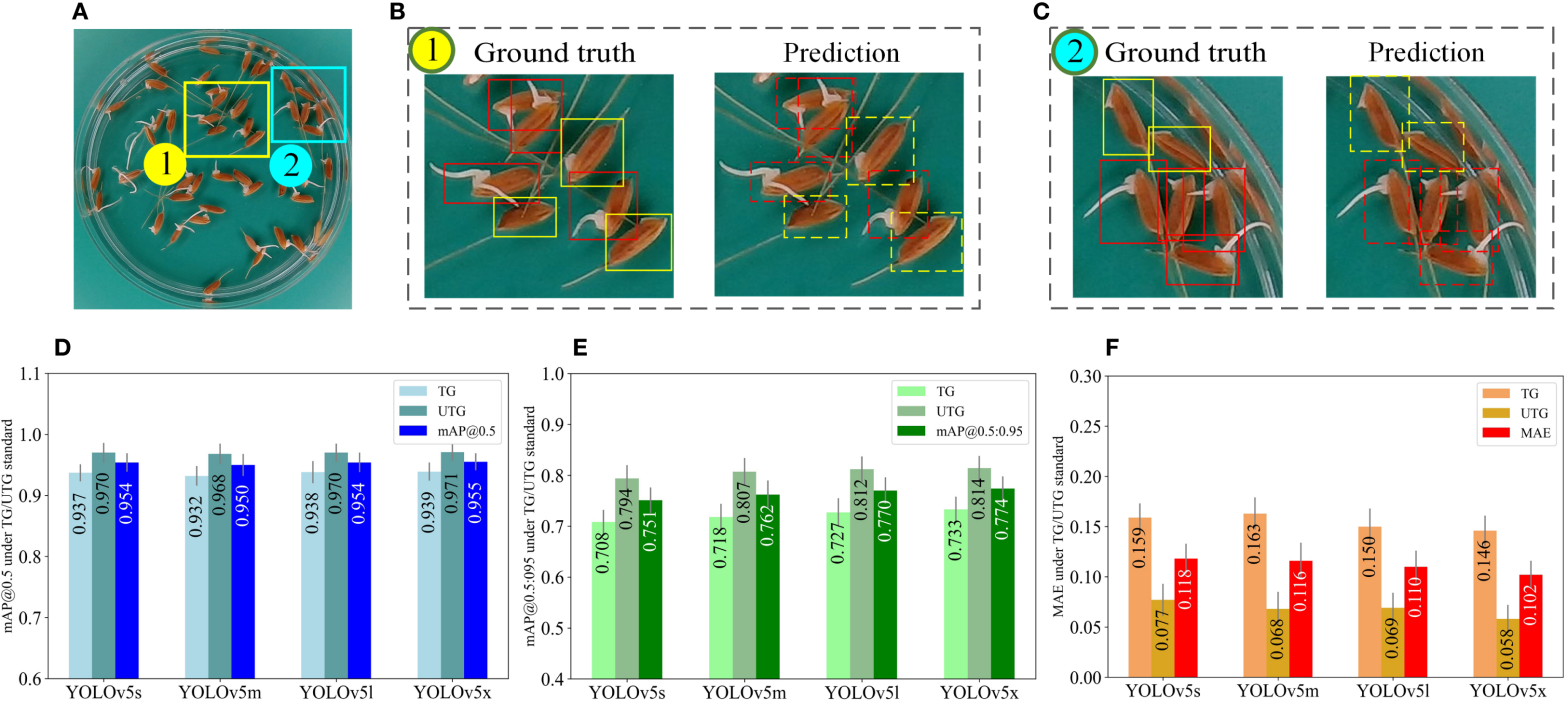
Model performance evaluation under the TG/UTG standard. **(A)** is a germinated seed image. The rectangular boxes in **(B, C)** indicate the ground truth (left) and the predicted results of YOLOv5m (right), where the red boxes indicate the germinated seeds and the green boxes indicate the ungerminated seeds. **(D–F)** represent the mAP@0.5, mAP@0.5:0.95, and MAE values obtained from testing 240 seed images using four YOLOv5 models (YOLOv5s, YOLOv5m, YOLOv5l, YOLOv5x), respectively (three decimal places are preserved), where error bar represents standard deviation.

To address these issues, we proposed a new seed germination standard TG/UTG, which considers seed radicle length greater than 2mm as an indicator of germination. As part of our experiments, we re-labeled the input images according to TG/UTG and evaluated the performance of the model. The model performance under the TG/UTG germination standard is shown in **Figure 8**. Panel (a) displays a representative image of germinated seeds, while (b) and (c) compare the ground truth annotations (left) with the predictions of the YOLOv5m model (right), where red boxes denote germinated seeds and green boxes denote ungerminated seeds. Panels (d), (e), and (f) present the evaluation metrics—mAP@0.5, mAP@0.5:0.95, and MAE—calculated on 240 test images using four YOLOv5 model variants (YOLOv5s, YOLOv5m, YOLOv5l, YOLOv5x). All results are reported with three decimal places, and error bars indicate the standard deviation, highlighting the performance stability of each model under the TG/UTG standard.

Upon adopting the TG/UTG germination standard, all four models have improved in mAP and MAE. In comparison with the results depicted in **Figure 7D**, the mAP@0.5 values of the four models (**Figure 8D**) improved by 0.012, 0.015, 0.020, and 0.020, respectively, while the MAE values (**Figure 8F**) decreased by 0.066, 0.048, 0.046, and 0.065, respectively. As shown in **Figure 8F**, all four models for ungerminated seeds have MAE values below 0.08mm. The main reason is that the germination standard of 2mm improves the distinction between germinated and ungerminated seeds, resulting in more accurate seed labeling.

To evaluate the accuracy of SeedRuler-YOLO, we conducted the following experiments. Firstly, the seeds were categorized into two types according to their germination speed: fast and slow germination. For the seeds with fast germination speed, after the seeds were placed in the constant temperature incubator, we measured the germination rate at 48 hours and 60 hours, respectively. For the seeds with slow germination speed, the measured time was set to 60 hours and 72 hours, respectively.

The selection of 48h, 60h, and 72h as germination time points was based on both biological and experimental considerations. These time intervals reflect distinct phases of rice seed germination, during which phenotypic variation becomes increasingly pronounced. This variation is conducive to capturing diverse germination behaviors and enables the identification of genes associated with stage-specific germination traits. Furthermore, this time frame was adopted by a recent high-impact Science study (Wei et al., 2024), which explored the genetic architecture of rice using over 18,000 lines. By following this standardized time scheme, our results gain both scientific rigor and comparability with large-scale genomic studies.

**Table 1** presents the results of measuring the germination rate for rice seeds of different six varieties: from ‘Type0’ to ‘Type5’. It can be seen from **Table 1** that, for seeds of six different varieties, SeedRuler-YOLO can accurately count the number of seeds. In addition, the relative error of the predicted germination rate predominantly falls within ±0.1. From **Additional File 1**: **Supplementary Figure S5**, we can observe that as the germination time increases and the germination degree becomes more pronounced, the accuracy of the germination rate also increases.

**Table 1:** The evaluation results of SeedRuler-YOLO.

Hybrid type	Parameter	Germination time								
		48 hours			60 hours			72 hours		
		Automatic count	Manual count	Relative error	Automatic count	Manual count	Relative error	Automatic count	Manual count	Relative error
Type0	No. of germinated seeds	22	20	-0.1	25	24	-0.04			
	No. of ungerminated seeds	12	14	0.14	9	10	0.10			
	No. of seeds	34	34	0	34	34	0			
	Germination rate	0.6471	0.5882	-0.10	0.7353	0.7059	-0.04			
Type1	No. of germinated seeds	43	43	0	43	43	0			
	No. of ungerminated seeds	0	0	0	0	0	0			
	No. of seeds	43	43	0	43	43	0			
	Germination rate	1	1	0	1	1	0			
Type2	No. of germinated seeds	25	23	-0.09	36	36	0			
	No. of ungerminated seeds	15	17	0.12	4	4	0			
	No. of seeds	40	40	0	40	40	0			
	Germination rate	0.625	0.575	-0.09	0.9	0.9	0			
Type3	No. of germinated seeds				25	24	-0.04	32	31	-0.03
	No. of ungerminated seeds				9	10	0.10	2	3	0.33
	No. of seeds				34	34	0	34	34	0
	Germination rate				0.7353	0.7059	-0.04	0.9412	0.9118	-0.03
Type4	No. of germinated seeds				34	34	0	37	37	0
	No. of ungerminated seeds				3	3	0	0	0	0
	No. of seeds				37	37	0	37	37	0
	Germination rate				0.9189	0.9189	0	1	1	0
Type5	No. of germinated seeds				23	23	0	29	29	0
	No. of ungerminated seeds				13	13	0	7	7	0
	No. of seeds				36	36	0	36	36	0
	Germination rate				0.6389	0.6389	0	0.8056	0.8056	0

A closer examination of **Table 1** reveals how changes in seed morphology over time influence detection accuracy. For fast-germinating varieties such as Type0 and Type2, the relative error at 60 hours is lower than at 48 hours, indicating improved detection performance as radicle elongation becomes more prominent. Type1 shows zero error at both time points, suggesting that for some lines with early and uniform germination, SeedRuler-YOLO can achieve high accuracy even at 48 hours. For slow-germinating varieties such as Type3, Type4, and Type5, detection at 60 hours and 72 hours yields accurate results, with relative errors at or close to zero. Notably, Type4 and Type5 exhibit zero error across both 60h and 72h, while Type3 shows comparable performance at both time points. These observations confirm that increased radicle visibility over time enhances the model’s ability to distinguish germinated seeds, and that SeedRuler-YOLO maintains robust performance across different germination dynamics.

### 3.4 Evaluation of SeedRuler-SAM

To further validate the segmentation performance of SeedRuler-SAM, we conducted a comparative experiment using two mainstream image segmentation models: Mask-RCNN (He et al., 2017) and UNet (Ronneberger et al., 2015). A subset of 100 test images was randomly selected, and all three methods were evaluated under the same conditions using four standard metrics: Dice, IoU, PA, and FPR.

As shown in **Table 2**, SeedRuler-SAM achieved the best performance across all metrics, with a Dice of 0.942, IoU of 0.841, PA of 0.916, and a FPR of only 0.011. In contrast, UNet achieved moderate results (Dice = 0.799, IoU = 0.697), and Mask-RCNN showed relatively lower accuracy (Dice = 0.723, IoU = 0.571). These results demonstrate the superior segmentation accuracy and reliability of SeedRuler-SAM in rice seed and radicle segmentation tasks.

**Table 2:** The metric values of the three algorithms.

	Dice↑	IoU↑	PA↑	FPR↓
Mask-RCNN	0.723±0.083	0.571±0.097	0.661±0.122	0.096±0.015
UNet	0.799±0.115	0.697±0.146	**0.942±0.032**	0.108±0.104
SeedRuler-SAM	**0.942±0.021**	**0.841±0.043**	0.916±0.024	**0.011±0.005**

↑ means higher is better; ↓ means lower is better. Bold values indicate best performance.

To verify the effectiveness of SeedRuler-SAM, we conducted germination assessment experiments on five images (**Additional File 1**: **Supplementary Figure S6**). Additionally, three seed images with varying radicle lengths were selected as germination reference standards (**Additional File 1**: **Supplementary Figure S7**), referred to as Criteria 1, Criteria 2, and Criteria 3, respectively. The assessment results, displayed in **Figure 4**, showcased a maximum deviation of only 2 units between the estimated values by SeedRuler-SAM and the ground truth. This outcome underscores the high accuracy and reliability of SeedRuler-SAM in estimating the germination rate.

## 4 Discussion

In this study, we developed a high-throughput phenotyping web server SeedRuler for the assessment of rice seed germination rate (Li et al., 2024b; Yao et al., 2024; Liu et al., 2025). SeedRuler utilizes deep learning and image processing technology for phenotyping seeds, which can provide large amounts of reliable data on time, reduce time and labor costs, and improve work efficiency.

Our experiments have highlighted the influence of several factors on the model performance: (1) Fast-germinating seeds exhibit a longer radicle during the specified germination period, resulting in a larger radicle area than the seed area (**Additional File 1**: **Supplementary Figure S8A**); (2) Denser seed distribution within the image increases the overlap of the manually labeled boxes (**Additional File 1**: **Supplementary Figure S8B**). SeedRuler has demonstrated its ability to mitigate the impact of these two factors on the model performance.

The image acquisition box was designed with a light source for capturing seed images (**Additional File 1**: **Supplementary Figure S1**), thereby improving the quality of the images, as well as allowing us to conduct experiments at any time of day, regardless of external environmental factors such as time and location. Experimental results indicate that image quality affects the performance of SeedRuler-YOLO. We have improved mAP@0.5 by 4-8% as well as mAP@0.5:0.95 by 10-30% and reduced MAE by 1-6% with our image acquisition box when compared to taking photos with a cell phone in natural light (**Additional File 1**: **Supplementary Table S2**) (Li et al., 2024c; Padmanabhan et al., 2025).

Additionally, we labeled the images with two different germination standards, OG/UOG and TG/UTG, to create two separate seed image datasets and trained the model individually for each case. Comparative analysis revealed that the choice of germination standard has a notable impact on model performance. When using the OG/UOG standard (1mm radicle length), the radicle is often too short to be clearly visible, resulting in higher labeling ambiguity and mediocre model performance. In contrast, adopting the TG/UTG standard (2mm radicle length) significantly improves model performance, reducing MAE by 4–7% (Klasen et al., 2025).

This effect is further illustrated by the performance results shown in **Figures 7** and **8**. **Figure 7** shows that under the OG/UOG standard, all four YOLOv5 models achieved lower mAP and higher MAE, particularly for ungerminated seeds. **Figure 8** demonstrates that switching to the TG/UTG standard leads to a clear improvement in both mAP and MAE across models. Among them, YOLOv5m consistently achieves the best balance between accuracy and speed. These figures emphasize the importance of selecting a biologically meaningful and visually distinguishable germination threshold.

Furthermore, we compared the results of identifying different varieties of rice seeds at various stages (over 48, 60, and 72 hours) of germination (**Additional File 1**: **Supplementary Figure S5**). The diverse shapes of the seeds including oval, oblate, oblong, among others, and varying seed coat colors such as yellow, black, red, etc., were considered. In addition, some seeds also featured rice awns while others did not. The experiments showed that the relative errors in the germination rate were within 0.1 for each image obtained using SeedRuler, and the errors in the number of germinated while errors in the counts of germinated and non-germinated within 0–2 seeds.

In addition, we have equipped SeedRuler-YOLO with a fixed germination standard, while SeedRuler-IP and SeedRuler-SAM offer users the option to define their own germination standards. SeedRuler-IP is based on image processing and offers the advantage that, if future updates are required, it eliminates the need for substantial manual annotation and training efforts. SeedRuler-SAM performs precise segmentation on the bounding boxes obtained from SeedRuler-YOLO using SAM, which is a pre-trained model that can be directly applied. Therefore, during the training of the SeedRuler model, only the YOLOv5 model needs to be trained. **Table 3** presents a comprehensive comparison of the characteristics of these three methods.

**Table 3:** Comparison of the characteristics of three methods: SeedRuler-IP, SeedRuler-YOLO, and SeedRuler-SAM.

Methods	SeedRuler-IP	SeedRuler-YOLO	SeedRuler-SAM
Methodology	K-means, morphological operations	YOLOv5	SAM
Supervised learning	×	✓	△
Train the model	×	✓	×
Mobile phone	✓	✓	✓
Dark background	✓	✓	✓
Seeds in contact	✓	✓	✓
Customized germination standard	✓	×	✓

‘√’ refers to able, ‘×’ refers to unable, and ‘△’ is partial.

SeedRuler is well-suited for the following different scenarios: (1) Seeds come in a variety of shapes and colors (**Additional File 1**: **Supplementary Figure S9**); (2) Radicles vary in length (**Additional File 1**: **Supplementary Figure S9**); (3) Petri dishes contain water droplets and reflections (**Additional File 1**: **Supplementary Figure S10**); (4) Seed images contain impurities such as rice awn and branch stalks (**Additional File 1**: **Supplementary Figure S10**). In summary, SeedRuler minimizes human intervention, offers ease and speed of use, and significantly improves work efficiency. Beyond the aforementioned capabilities, our future plans for SeedRuler encompass the integration of additional functionalities, including the detection of rice seed setting rate and chalkiness (Guo et al., 2021; Wang et al., 2022; Cai et al., 2024; Li et al., 2024a). These enhancements aim to augment and enrich the overall functionality of SeedRuler.

In addition, SeedRuler has demonstrated strong scalability and usability in practical applications. The platform supports both web-based and offline deployment, enabling flexible usage across various operating environments. The web server is capable of concurrent processing through GPU acceleration and MySQL-based data management, while the offline version supports cross-platform operation (Windows, macOS, Linux) with a graphical user interface developed in PyQt5. The user-friendly interface includes intuitive modules for image upload, germination detection, and result export, which reduces the technical barrier for end users. Furthermore, batch processing capabilities and customizable germination standards enhance its adaptability to large-scale phenotyping projects. These features collectively support our claims regarding the scalability and usability of SeedRuler.

It is important to note that the training and evaluation in this study were conducted using seed images captured with a standardized imaging box under consistent lighting and background conditions. While this setup ensures high image quality and stable model performance, it may limit the generalizability of the system when applied to images acquired in uncontrolled environments, such as those taken with mobile phones under natural light or varying backgrounds. To overcome this limitation, we recommend that users ensure sufficient and uniform lighting and reduce background noise during image acquisition. Additionally, retraining the YOLO model or fine-tuning the parameters using their own image datasets can help improve the robustness and adaptability of SeedRuler to diverse usage scenarios.

To further evaluate the performance of SeedRuler-YOLO against existing tools, we conducted a benchmarking experiment using SeedQuant (Braguy et al., 2021), a deep learning-based seed germination analysis tool. We randomly selected 10 groups from the test set, with each group consisting of 20 seed images. For each image, we calculated the germination rate using both SeedQuant and SeedRuler-YOLO, and then computed the absolute error between the predicted and ground truth germination rates. The average and standard deviation of the absolute errors were calculated for each group. The results, summarized in **Table 4**, show that SeedRuler-YOLO consistently achieved lower mean absolute errors across all groups. The overall average absolute error of SeedRuler-YOLO was 0.040±0.037, compared to 0.106±0.095 for SeedQuant, demonstrating superior accuracy and robustness. These results confirm that SeedRuler-YOLO outperforms SeedQuant in terms of precision and reliability under the same experimental conditions.

**Table 4:** Germination rate error comparison between SeedQuant and SeedRuler-YOLO.

Rice group	SeedQuant	SeedRuler-YOLO
1	0.065±0.037	0.024±0.022
2	0.131±0.141	0.044±0.059
3	0.093±0.092	0.045±0.043
4	0.121±0.131	0.054±0.033
5	0.077±0.053	0.052±0.036
6	0.114±0.076	0.034±0.040
7	0.141±0.121	0.034±0.036
8	0.075±0.057	0.063±0.040
9	0.162±0.102	0.023±0.012
10	0.083±0.069	0.031±0.029
Total	0.106±0.095	0.040±0.037

This robustness can be attributed, in part, to the diversity of the training dataset. The rice resources used in this study were derived from nine genetically diverse populations cultivated in the experimental field of Shanghai Normal University (Xuehui Huang Lab). These seeds exhibit substantial phenotypic variation in grain morphology, including differences in length, shape, and color. As a result, the constructed dataset covers a wide range of genotypes and visual characteristics. This diversity enables the trained SeedRuler-YOLO model to generalize well across various seed types and accurately detect germinated seeds regardless of morphological differences such as seed shape or radicle length and color. Moreover, since all images were collected using a standardized imaging box with a uniform background and consistent lighting conditions, the influence of background variation on model performance is minimized. However, in non-standard environments with complex or inconsistent backgrounds, the model’s detection accuracy may be affected. Therefore, we recommend retraining or fine-tuning the model when applying it to datasets acquired under different imaging conditions.

To address the concern regarding error handling and robustness, we conducted additional experiments using six representative images that simulate various complex or extreme scenarios commonly encountered in practical seed germination tests. These scenarios include: (1) shadows and mirrored seed placements, (2) severe seed overlapping and contact, (3) seeds with awns, (4) seeds with elongated radicles, (5) background text interference, and (6) entangled radicles and awns.

As shown in **Additional File 1**: **Supplementary Figure S11**, SeedRuler-YOLO consistently achieved accurate detection results across all conditions. Germinated seeds were correctly identified and marked with cyan boxes, while non-germinated seeds were marked with blue boxes. These results demonstrate the model’s robustness in handling occlusion, deformation, noise interference, and morphological complexity. The performance under such scenarios indicates that SeedRuler-YOLO possesses strong adaptability and stability, minimizing missegmentation and omission rates even in extreme cases. This robustness is crucial for ensuring reliability in real-world germination analysis tasks.

Despite SeedRuler’s user-friendly design and strong performance under controlled conditions, several limitations remain that may impact its broader applicability. Notably, the current system abstracts away most algorithm-level configurations, which limits expert users from tailoring detection models or segmentation thresholds to specialized datasets or experimental goals. While this design choice facilitates ease of adoption, it may restrict flexibility in advanced research scenarios that require fine-tuned control over detection sensitivity or model behavior.

Additionally, although the image acquisition box helps standardize lighting and imaging angles, the platform’s performance may decline when analyzing images taken under natural light, complex backgrounds, or with varying camera devices—conditions often encountered in field-based or decentralized experiments. This sensitivity to imaging conditions highlights the need for more robust pre-processing pipelines or adaptive models in future versions.

Moreover, SeedRuler does not currently support advanced automation features such as scheduled batch analysis, task queue management, or background processing. These capabilities are commonly found in platforms like Transmission or qBittorrent and are particularly valuable in high-throughput phenotyping workflows. Incorporating such features would enhance the platform’s scalability and usability in large-scale or unattended processing environments.

## 5 Conclusion

In this study, we developed and validated SeedRuler, a web-based, high-throughput platform for assessing rice seed germination. By integrating three complementary modules—SeedRuler-YOLO (deep learning-based detection), SeedRuler-SAM (interactive segmentation using the Segment Anything Model), and SeedRuler-IP (traditional image processing)—the system offers high accuracy, flexibility, and broad usability. SeedRuler can be accessed at (http://www.xhhuanglab.cn/tool/SeedRuler.html).

Extensive experiments demonstrated that SeedRuler-YOLO achieved a mean average precision (mAP@0.5) of 0.955 and a mean absolute error (MAE) of 0.110. Meanwhile, SeedRuler-SAM outperformed baseline segmentation models in terms of Dice and IoU. Importantly, the platform supports both fixed and user-defined germination standards, batch processing, and cross-platform deployment (online and offline), making it accessible to a wide range of users.

Despite its strong performance, SeedRuler has limitations, such as the lack of advanced automation features (e.g., task scheduling and background processing) and sensitivity to image quality in uncontrolled environments. These issues will be addressed in future versions through improved pre-processing, parameter customization, and automation tools.

Overall, SeedRuler fills a critical gap in current seed phenotyping tools by offering an intuitive yet powerful solution for germination analysis. It is expected to accelerate breeding programs, facilitate genetic research, and contribute to the broader advancement of AI-driven agricultural technologies.

## Data Availability

The datasets presented in this study can be found in online repositories. The names of the repository/repositories and accession number(s) can be found below: https://www.kaggle.com/jinfengzhao/riceseedgermination.

## References

[B1] Adli T. Bujaković D. Bondžulić B. Laidouni M. Z. Andrić M. (2025). A modified yolov5 architecture for aircraft detection in remote sensing images. J. Indian Soc. Remote Sens. 53, 933–948. doi: 10.1007/s12524-024-02033-7

[B2] Awty-Carroll D. Clifton-Brown J. Robson P. (2018). Using k-nn to analyse images of diverse germination phenotypes and detect single seed germination in miscanthus sinensis. Plant Methods 14, 1–7. doi: 10.1186/s13007-018-0272-0, PMID: 29371877 PMC5771004

[B3] Braguy J. Ramazanova M. Giancola S. Jamil M. Kountche B. A. Zarban R.. (2021). Seedquant: A deep learning-based tool for assessing stimulant and inhibitor activity on root parasitic seeds. Plant Physiol. 186, 1632–1644. doi: 10.1093/plphys/kiab173, PMID: 33856485 PMC8260127

[B4] Cai Z. Liu X. Ning Z. Wang S. Li D. (2024). “Automated three-dimensional non-destructive detection of rice chalkiness driven by vgg-unet,” in 2024 9th International Conference on Intelligent Computing and Signal Processing (ICSP). Proceeding of the International Conference on Intelligent Computing and Signal Processing (ICSP). Xian, China: IEEE. p. 1722–1726.

[B5] Chai H. H. Lu Y. Fang C. Li Y. D. Kang Y. J. Li C. M.. (2018). 3d-printed seed planter and well array for high-throughput seed germination screening. Integr. Biol. 10, 67–73. doi: 10.1039/C7IB00178A, PMID: 29234768

[B6] Chaivivatrakul S. (2020). Automatic assessment of seed germination percentage. Eng. J. 24, 85–96. doi: 10.4186/ej.2020.24.4.85

[B7] Colmer J. O’Neill C. M. Wells R. Bostrom A. Reynolds D. Websdale D.. (2020). Seedgerm: A cost-effective phenotyping platform for automated seed imaging and machine-learning based phenotypic analysis of crop seed germination. New Phytol. 228, 778–793. doi: 10.1111/nph.16736, PMID: 32533857

[B8] Dheeraj A. Chand S. (2025). Deep learning based weed classification in corn using improved attention mechanism empowered by explainable ai techniques. Crop Prot. 190, 107058. doi: 10.1016/j.cropro.2024.107058

[B9] ElMasry G. Mandour N. Wagner M.-H. Demilly D. Verdier J. Belin E.. (2019). Utilization of computer vision and multispectral imaging techniques for classification of cowpea (vigna unguiculata) seeds. Plant Methods 15, 1–16. doi: 10.1186/s13007-019-0411-2, PMID: 30911323 PMC6417027

[B10] Genze N. Bharti R. Grieb M. Schultheiss S. J. Grimm D. G. (2020). Accurate machine learning-based germination detection, prediction and quality assessment of three grain crops. Plant Methods 16, 1–11. doi: 10.1186/s13007-020-00699-x, PMID: 33353559 PMC7754596

[B11] Guo Y. Li S. Zhang Z. Li Y. Hu Z. Xin D.. (2021). Automatic and accurate calculation of rice seed setting rate based on image segmentation and deep learning. Front. Plant Sci. 12, 770916. doi: 10.3389/fpls.2021.770916, PMID: 34970287 PMC8712771

[B12] He K. Gkioxari G. Dollár P. Girshick R. (2017). “Mask r-cnn,” in Proceedings of the IEEE international conference on computer vision. Proceedings of the IEEE International Conference on Computer Vision (ICCV). Venice, Italy: IEEE. p. 2961–2969.

[B13] Huang Z. Wang J. Fu X. Yu T. Guo Y. Wang R. (2020). Dc-spp-yolo: Dense connection and spatial pyramid pooling based yolo for object detection. Information Sci. 522, 241–258.

[B14] Joosen R. V. Kodde J. Willems L. A. Ligterink W. van der Plas L. H. Hilhorst H. W. (2010). Germinator: A software package for high-throughput scoring and curve fitting of arabidopsis seed germination. Plant J. 62, 148–159. doi: 10.1111/j.1365-313X.2009.04116.x, PMID: 20042024

[B15] Khoenkaw P. (2016). “An image-processing based algorithm for rice seed germination rate evaluation,” in 2016 International Computer Science and Engineering Conference (ICSEC). 1–5, IEEE.

[B16] Kirillov A. Mintun E. Ravi N. Mao H. Rolland C. Gustafson L.. (2023). Segment anything. arXiv preprint arXiv:2304.02643.

[B17] Klasen D. Fischbach A. Sydoruk V. Kochs J. Bühler J. Koller R.. (2025). Seed-to-plant-tracking: Automated phenotyping of seeds and corresponding plants of arabidopsis. Front. Plant Sci. 16, 1539424. doi: 10.3389/fpls.2025.1539424, PMID: 40357151 PMC12066762

[B18] Li X. Hou B. Tang H. Talpur B. A. Zeeshan Z. Bhatti U. A.. (2024c). Abnormal crops image data acquisition strategy by exploiting edge intelligence and dynamic-static synergy in smart agriculture. Proceedings of the International Computer Science and Engineering Conference (ICSEC). Chiang Mai, Thailand: IEEE. p. 12538–12553. doi: 10.1109/JSTARS.2024.3414306

[B19] Li B. Huang A. Wang L. Wu S. Xu Z. Chen X.. (2024a). Increased sugar content impairs pollen fertility and reduces seed-setting in high-photosynthetic-efficiency rice. Crop J. 12, 1547–1558. doi: 10.1016/j.cj.2024.09.016

[B20] Li H. Liu L. Li Q. Liao J. Liu L. Zhang Y.. (2024b). Rsg-yolov8: Detection of rice seed germination rate based on enhanced yolov8 and multi-scale attention feature fusion. PLoS One 19, e0306436. doi: 10.1371/journal.pone.0306436, PMID: 39531481 PMC11556723

[B21] Liu L. Gao Q. Zhou J. (2025). Research on high-efficiency rice germination recognition algorithm based on image processing. Acad. J. Computing Inf. Sci. 8, 11–16. doi: 10.25236/AJCIS.2025.080702

[B22] Maleki H. H. Darvishzadeh R. Azad N. (2025). Sweet pepper yield modeling via deep learning and selection of superior genotypes using gblup and mgidi. Sci. Rep. 15, 1–12. doi: 10.1038/s41598-025-99779-y, PMID: 40289216 PMC12034786

[B23] Padmanabhan V. N. Deekshitha D. S. P. Joseph M. N. (2025). “Ai-driven plant classification and cultivation optimization using high-throughput image acquisition for plant phenotyping,” in 2025 7th International Conference on Inventive Material Science and Applications (ICIMA). Proceedings of the International Conference on Inventive Material Science and Applications (ICIMA). Namakkal, India: IEEE. p. 1184–1190.

[B24] Pouvreau J.-B. Gaudin Z. Auger B. Lechat M.-M. Gauthier M. Delavault P.. (2013). A high-throughput seed germination assay for root parasitic plants. Plant Methods 9, 1–12. doi: 10.1186/1746-4811-9-32, PMID: 23915294 PMC3751143

[B25] Rajalakshmi R. Faizal S. Sivasankaran S. Geetha R. (2024). Riceseednet: Rice seed variety identification using deep neural network. J. Agric. Food Res. 16, 101062. doi: 10.1016/j.jafr.2024.101062

[B26] Redmon J. Divvala S. Girshick R. Farhadi A. (2016). “You only look once: Unified, real-time object detection,” in Proceedings of the IEEE conference on computer vision and pattern recognition. Proceedings of the IEEE Conference on Computer Vision and Pattern Recognition (CVPR). Las Vegas, NV, USA: IEEE. p. 779–788.

[B27] Redmon J. Farhadi A. (2017). “Yolo9000: Better, faster, stronger,” in Proceedings of the IEEE conference on computer vision and pattern recognition. Proceedings of the IEEE Conference on Computer Vision and Pattern Recognition (CVPR). Honolulu, HI, USA: IEEE. p. 7263–7271.

[B28] Rezaei M. Diepeveen D. Laga H. Gupta S. Jones M. G. Sohel F. (2025). A transformer-based few-shot learning pipeline for barley disease detection from field-collected imagery. Comput. Electron. Agric. 229, 109751. doi: 10.1016/j.compag.2024.109751

[B29] Ronneberger O. Fischer P. Brox T. (2015). “U-net: Convolutional networks for biomedical image segmentation,” in International Conference on Medical image computing and computer-assisted intervention. Medical Image Computing and Computer-Assisted Intervention – MICCAI 2015 (MICCAI 2015). Munich, Germany: IEEE. p. 234–241.

[B30] Sangjan W. Kick D. R. Washburn J. D. (2025). Improving plant breeding through ai-supported data integration. Theor. Appl. Genet. 138, 132. doi: 10.1007/s00122-025-04910-2, PMID: 40455285

[B31] Sindagi V. A. Patel V. M. (2018). A survey of recent advances in cnn-based single image crowd counting and density estimation. Pattern Recognition Lett. 107, 3–16. doi: 10.1016/j.patrec.2017.07.007

[B32] Su M. Ma Y. Zhang X. Wang Y. Zhang Y. (2017). Mbr-sift: A mirror reflected invariant feature descriptor using a binary representation for image matching. PLoS One 12, e0178090. doi: 10.1371/journal.pone.0178090, PMID: 28542537 PMC5436860

[B33] Tang Y. Huang W. Tan Z. Chen W. Wei S. Zhuang J.. (2025). Citrus fruit detection based on an improved yolov5 under natural orchard conditions. Int. J. Agric. Biol. Eng. 18, 176–185. doi: 10.25165/j.ijabe.20251803.8935

[B34] Urena R. Rodrıguez F. Berenguel M. (2001). A machine vision system for seeds quality evaluation using fuzzy logic. Comput. Electron. Agric. 32, 1–20. doi: 10.1016/S0168-1699(01)00150-8

[B35] Viola P. Jones M. (2001). “Rapid object detection using a boosted cascade of simple features,” in Proceedings of the 2001 IEEE computer society conference on computer vision and pattern recognition. CVPR 2001. Proceedings of the 2001 IEEE Computer Society Conference on Computer Vision and Pattern Recognition. CVPR 2001. Kauai, HI, USA: IEEE. p. I–I.

[B36] Wang C. Caragea D. Kodadinne Narayana N. Hein N. T. Bheemanahalli R. Somayanda I. M.. (2022). Deep learning based high-throughput phenotyping of chalkiness in rice exposed to high night temperature. Plant Methods 18, 9. doi: 10.1186/s13007-022-00839-5, PMID: 35065667 PMC8783510

[B37] Wang N. Fu S. Rao Q. Zhang G. Ding M. (2025). Insect-yolo: A new method of crop insect detection. Comput. Electron. Agric. 232, 110085. doi: 10.1016/j.compag.2025.110085

[B38] Waseem M. Sajjad M. M. Naqvi L. H. Majeed Y. Rehman T. U. Nadeem T. (2025). Deep learning model for precise and rapid prediction of tomato maturity based on image recognition. Food Phys. 2, 100060. doi: 10.1016/j.foodp.2025.100060

[B39] Wei X. Chen M. Zhang Q. Gong J. Liu J. Yong K.. (2024). Genomic investigation of 18,421 lines reveals the genetic architecture of rice. Science 385, eadm8762. doi: 10.1126/science.adm8762, PMID: 38963845

[B40] Yao Q. Zheng X. Zhou G. Zhang J. (2024). Sgr-yolo: A method for detecting seed germination rate in wild rice. Front. Plant Sci. 14, 1305081. doi: 10.3389/fpls.2023.1305081, PMID: 38322421 PMC10844399

[B41] Zhao J. Ma Y. Yong K. Zhu M. Wang Y. Luo Z.. (2023a). Deep-learning-based automatic evaluation of rice seed germination rate. J. Sci. Food Agric. 103, 1912–1924. doi: 10.1002/jsfa.12318, PMID: 36335532

[B42] Zhao J. Ma Y. Yong K. Zhu M. Wang Y. Wang X.. (2023b). Rice seed size measurement using a rotational perception deep learning model. Comput. Electron. Agric. 205, 107583. doi: 10.1016/j.compag.2022.107583

[B43] Zhou J. Applegate C. Alonso A. D. Reynolds D. Orford S. Mackiewicz M.. (2017). Leaf-gp: An open and automated software application for measuring growth phenotypes for arabidopsis and wheat. Plant Methods 13, 1–17. doi: 10.1186/s13007-017-0266-3, PMID: 29299051 PMC5740932

