# Supplementary material for: New-generation rice seed germination assessment: high efficiency and flexibility via SeedRuler web-based platform
Source: Front Plant Sci. 2025 Oct 13;16:1671998. doi: 10.3389/fpls.2025.1671998 (PMC12554718; doi:10.3389/fpls.2025.1671998)
Supplement: Supplementary file 1 [file DataSheet1.docx]

**New-generation Rice Seed Germination Assessment: High Efficiency and Flexibility via SeedRuler Web-based Platform**

Zeyu Hou^1^, Jinfeng Zhao^2^, Sheng Dai^1^, Jiawen Yang^1^, Yan Ma^1^, Ming Gong ^3,*^

1 College of Information, Mechanical and Electrical Engineering, Shanghai Normal University, Shanghai 200234, China

2 Shanghai Key Laboratory of Plant Molecular Sciences, College of Life Sciences, Shanghai Normal University, Shanghai 200234, China

3 School of Education, Shanghai Normal University, Shanghai 200234, China

^*^ Correspondence: ming@shnu.edu.cn


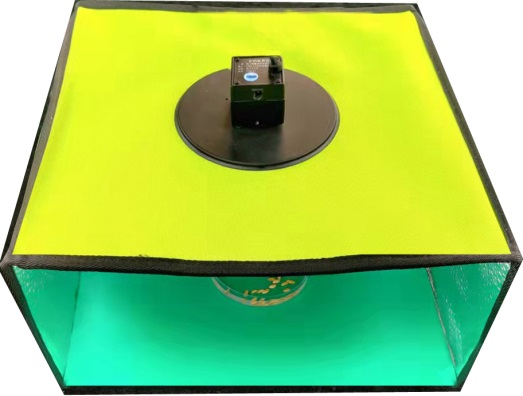

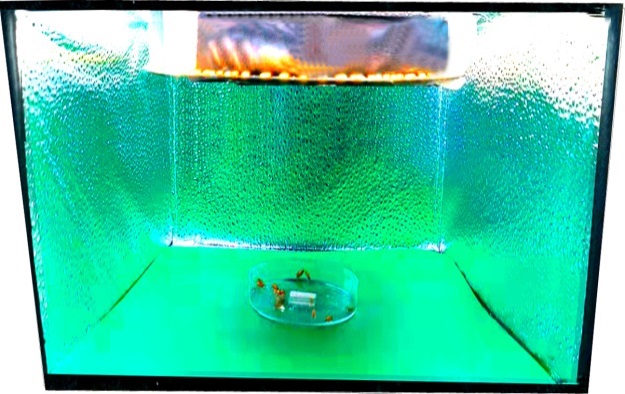


(a) (b)

Supplemental Figure S1 The rice seed image acquisition box: (a) Top view, (b) Front view.


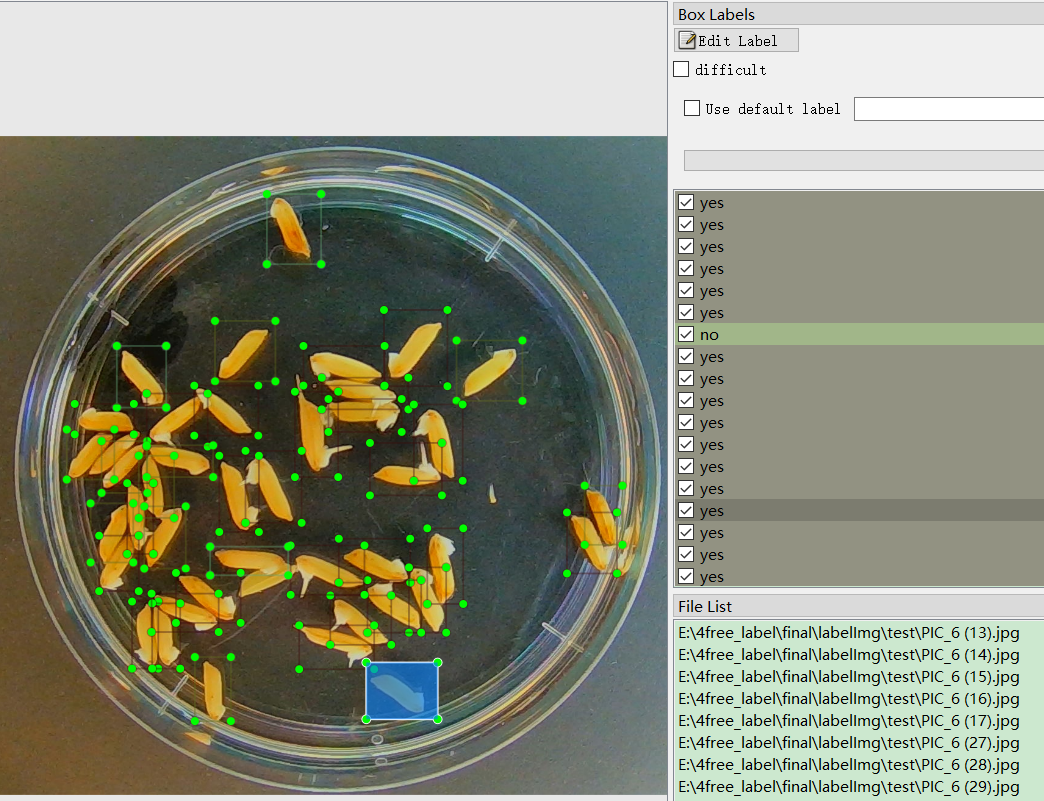


Supplemental Figure S2 The results of labeling with LabelImg.


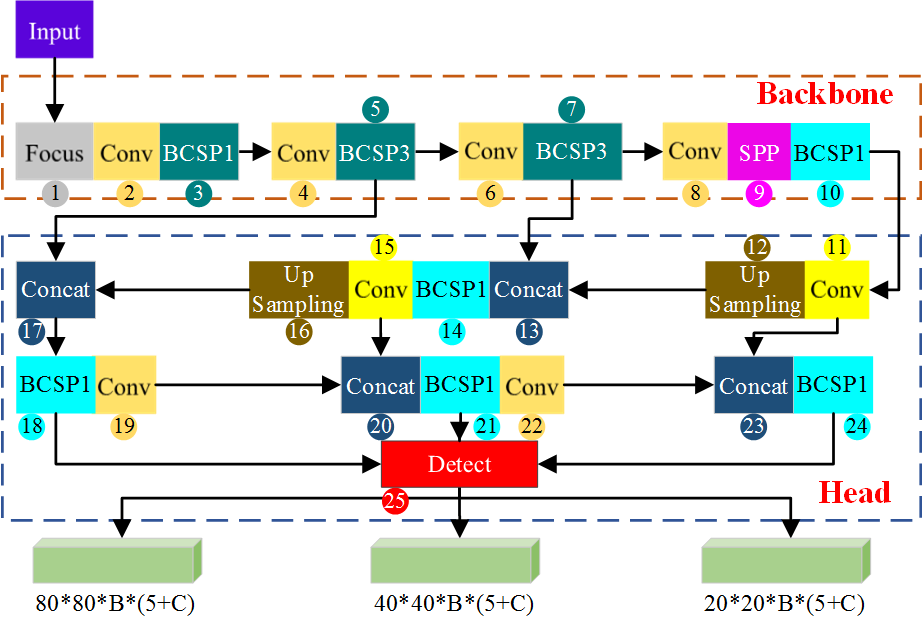

Supplemental Figure S3 Network architecture of YOLOv5.


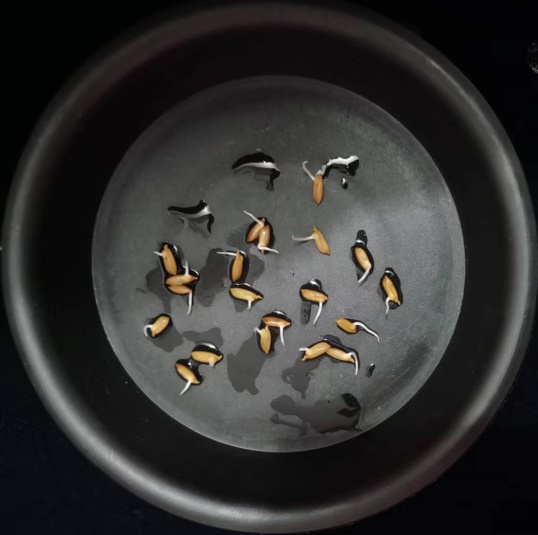

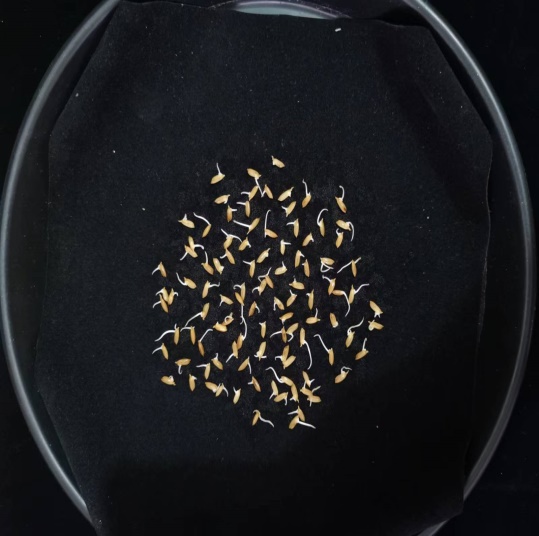

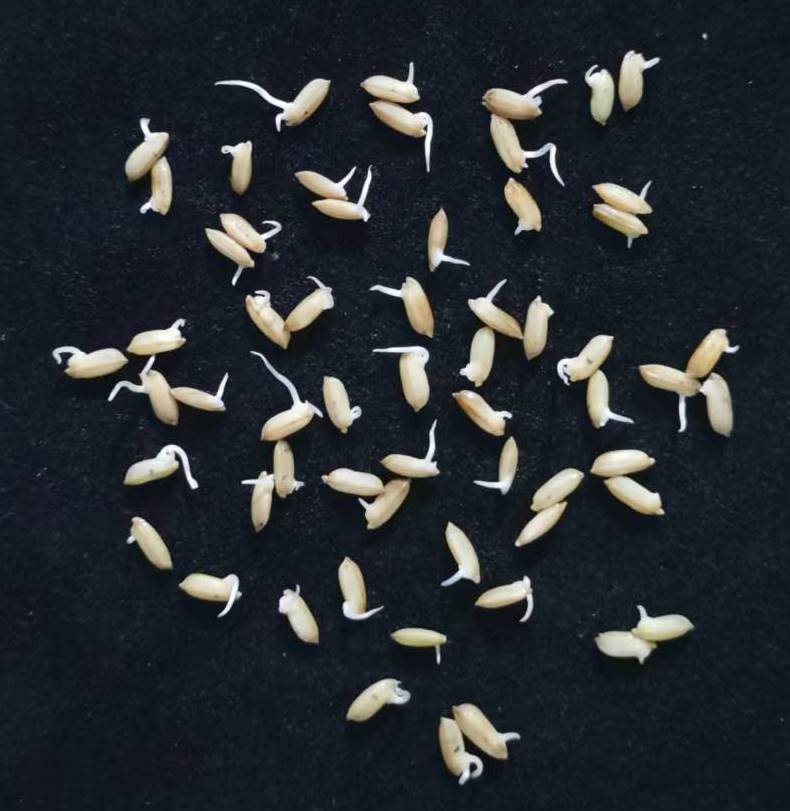

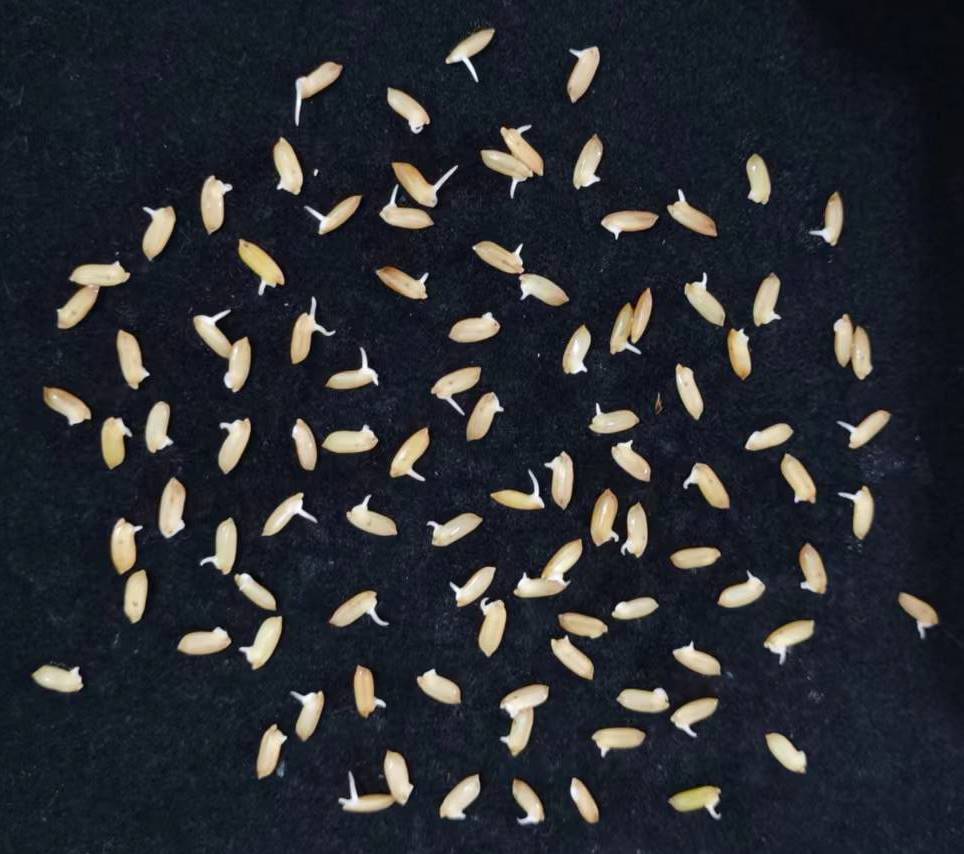


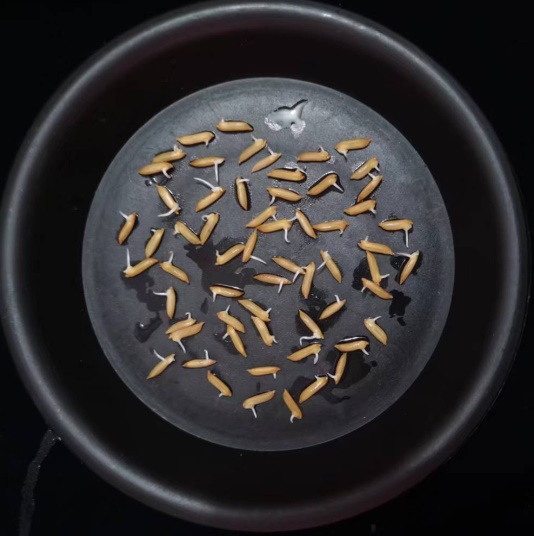

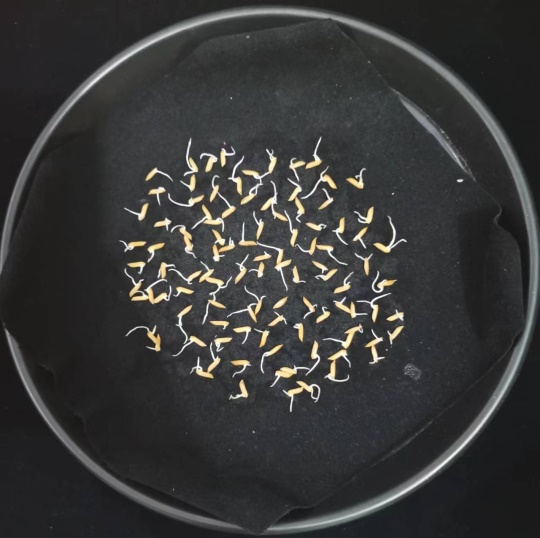


Supplemental Figure S4 Six sample images.


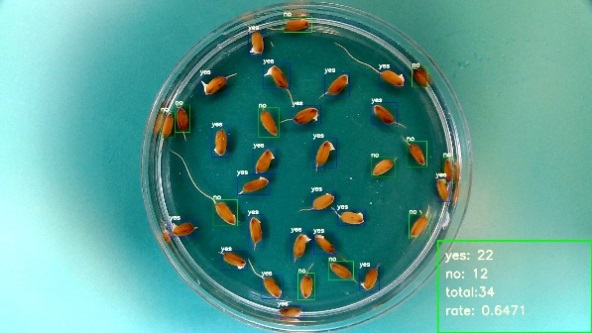

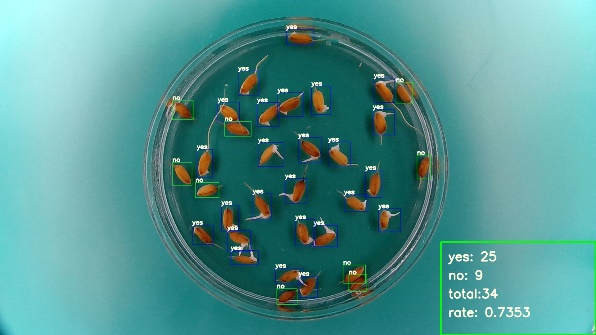


Type0 (after 48hours) Type0 (after 60 hours)


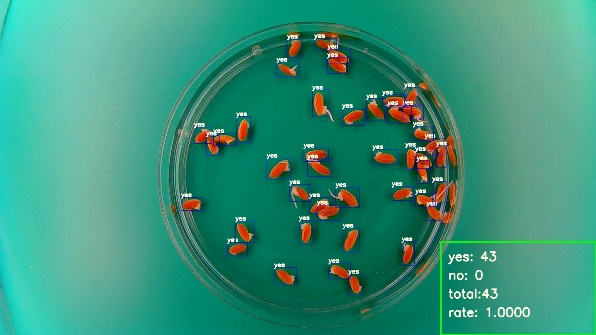

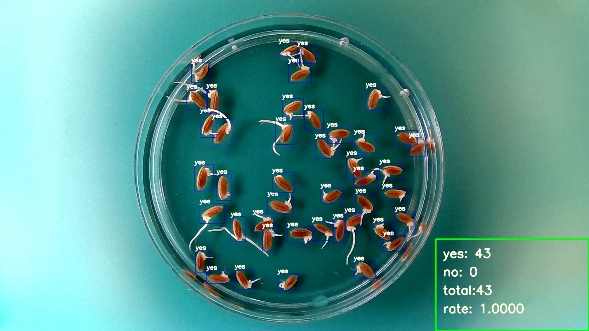


Type1 (after 48 hours) Type1 (after 60 hours)


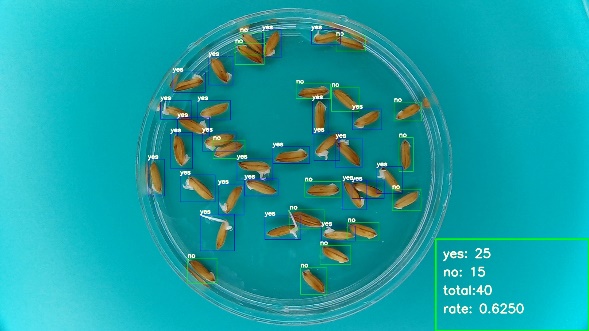

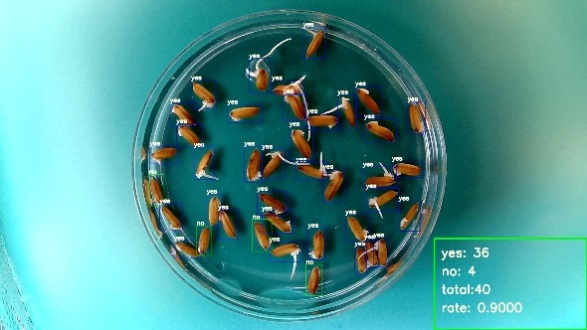


Type2 (after 48 hours) Type2 (after 60 hours)


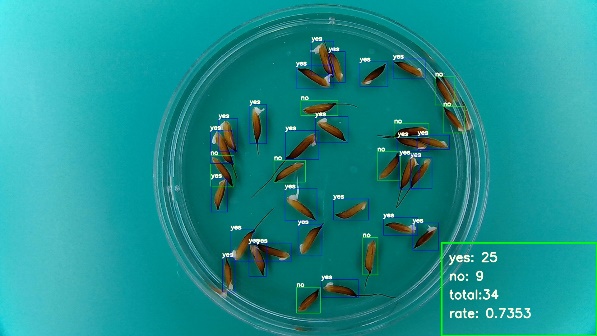

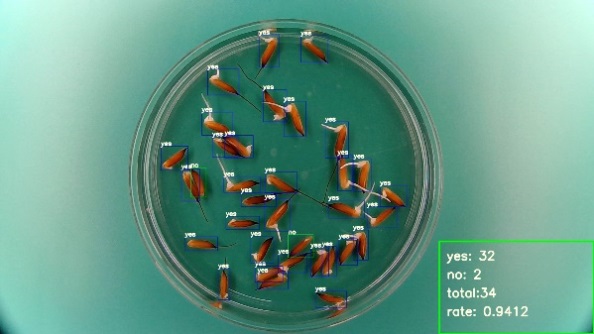


Type3 (after 60 hours) Type3 (after 72 hours)


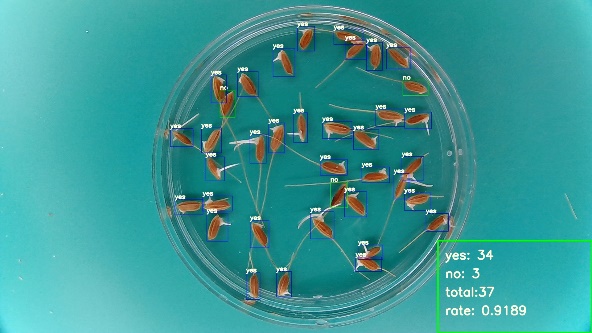

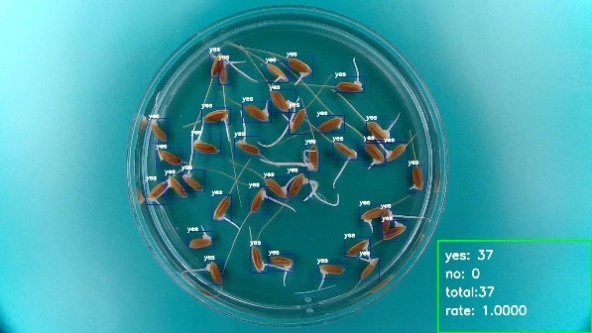


Type4 (after 60 hours) Type4 (after 72 hours)


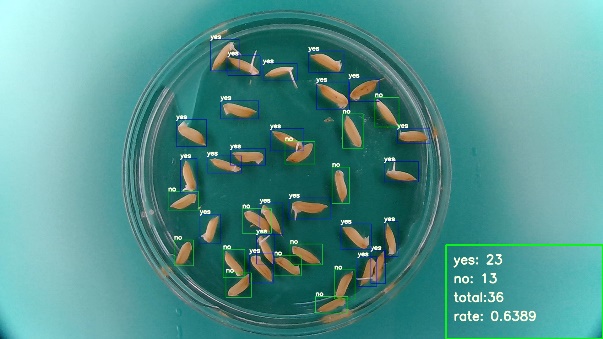

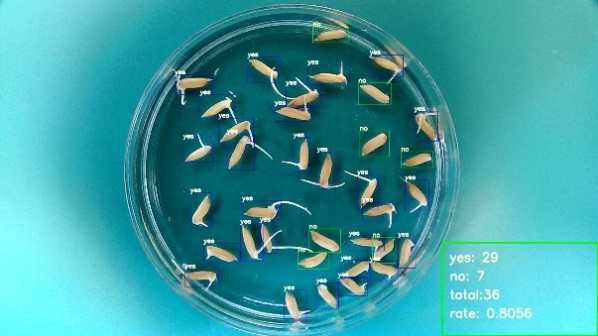


Type5 (after 60 hours) Type5 (after 72 hours)

Supplemental Figure S5 The evaluation results of SeedRuler-YOLO.


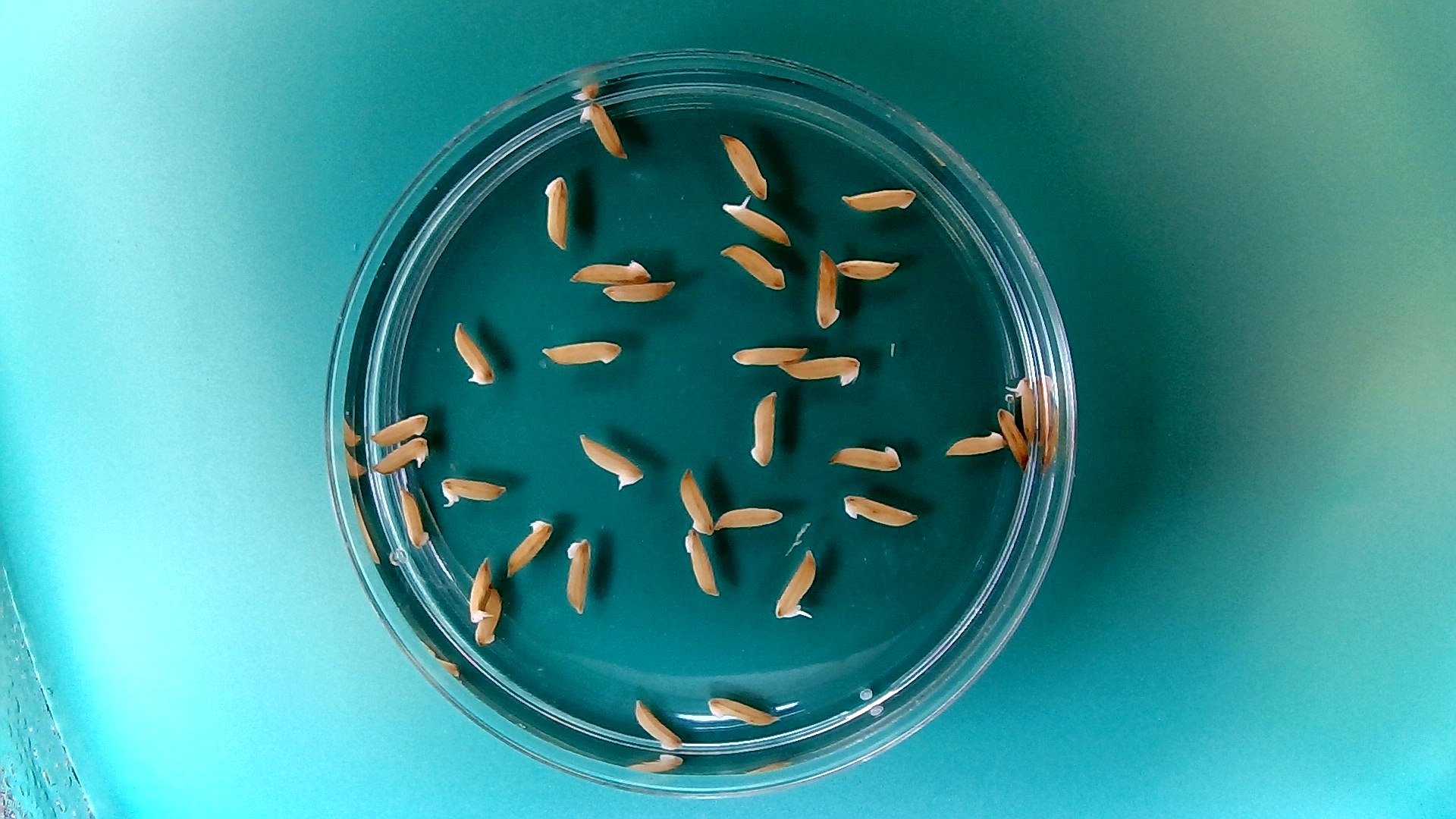

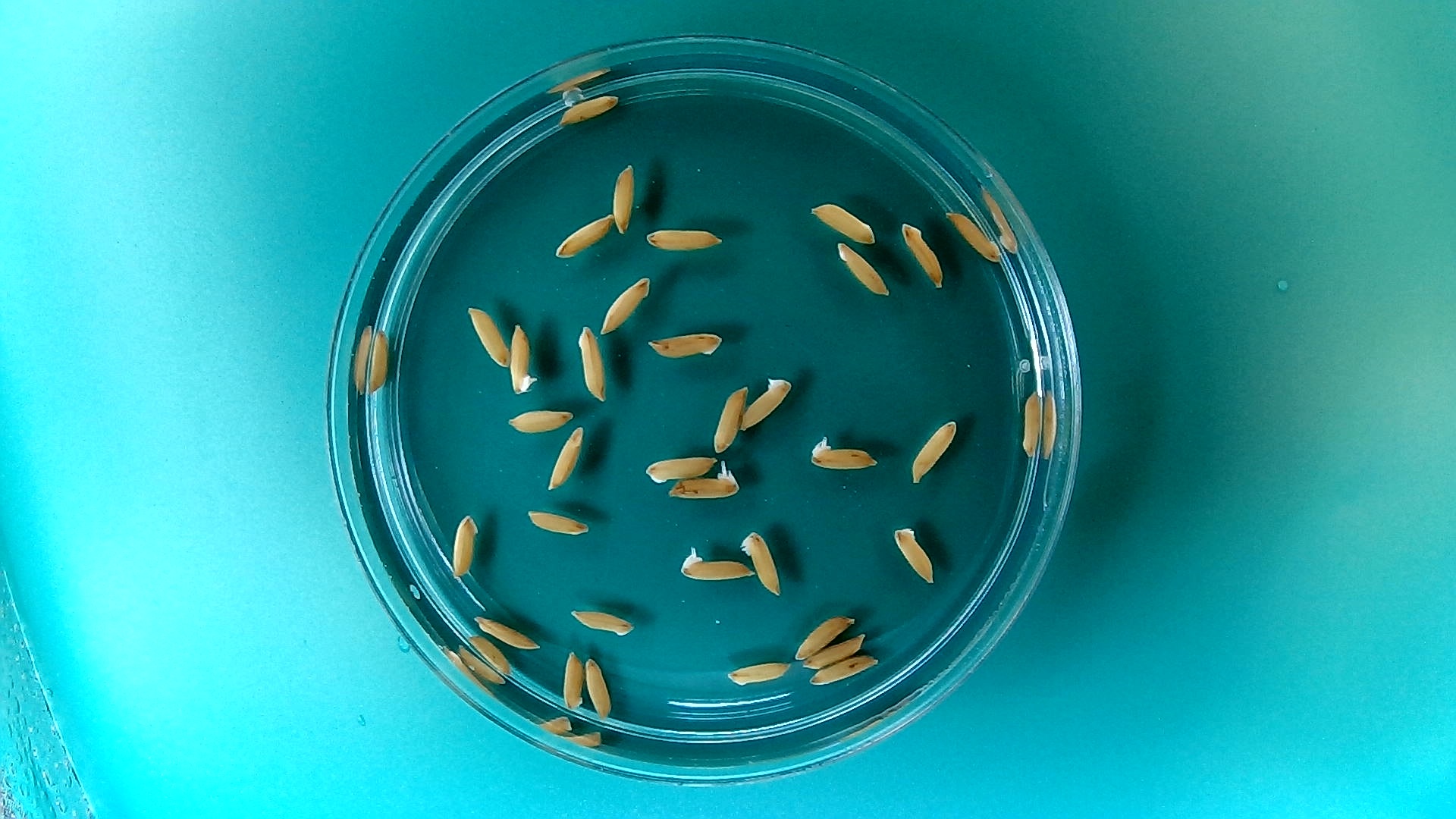


I1 I2


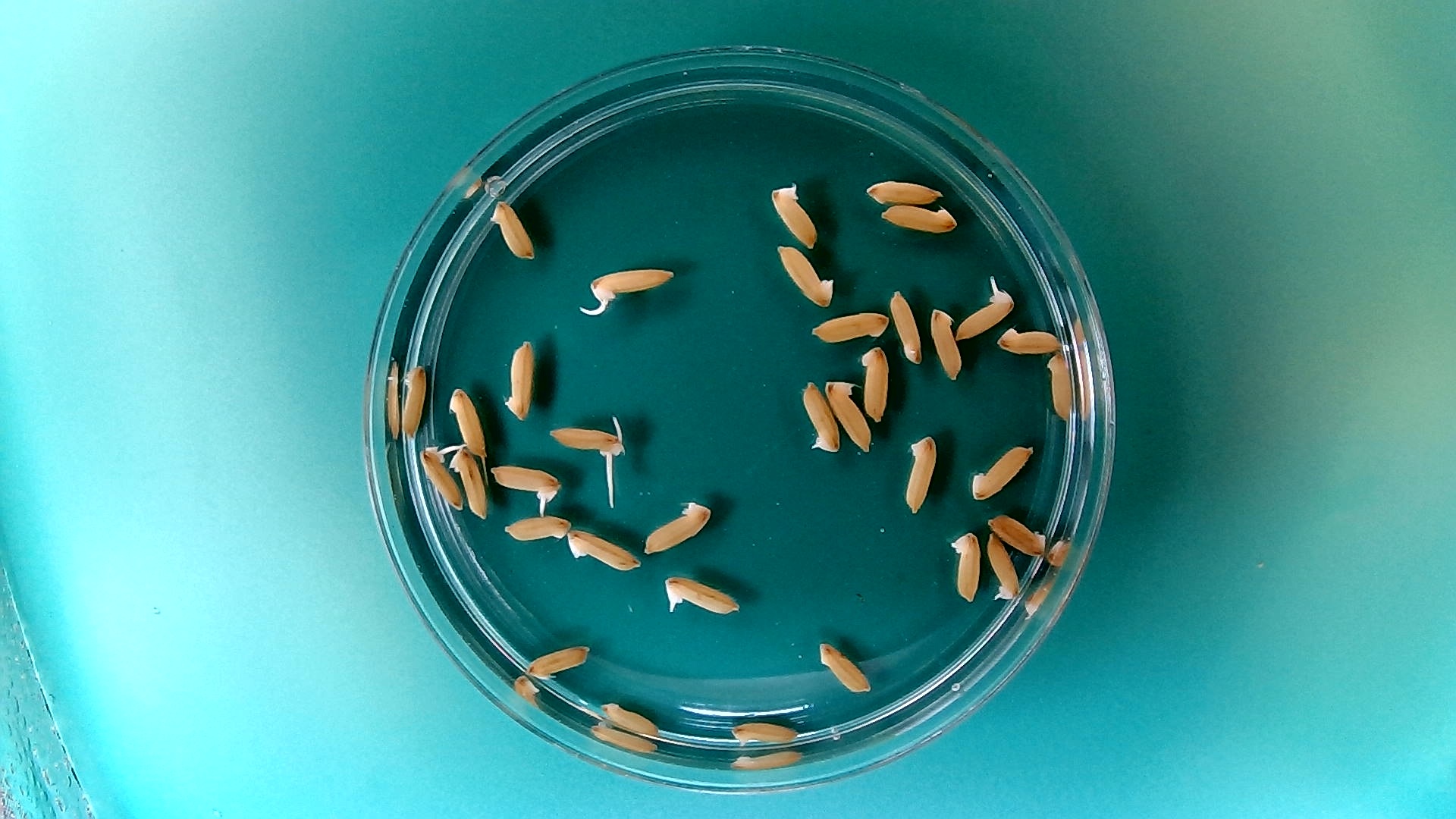

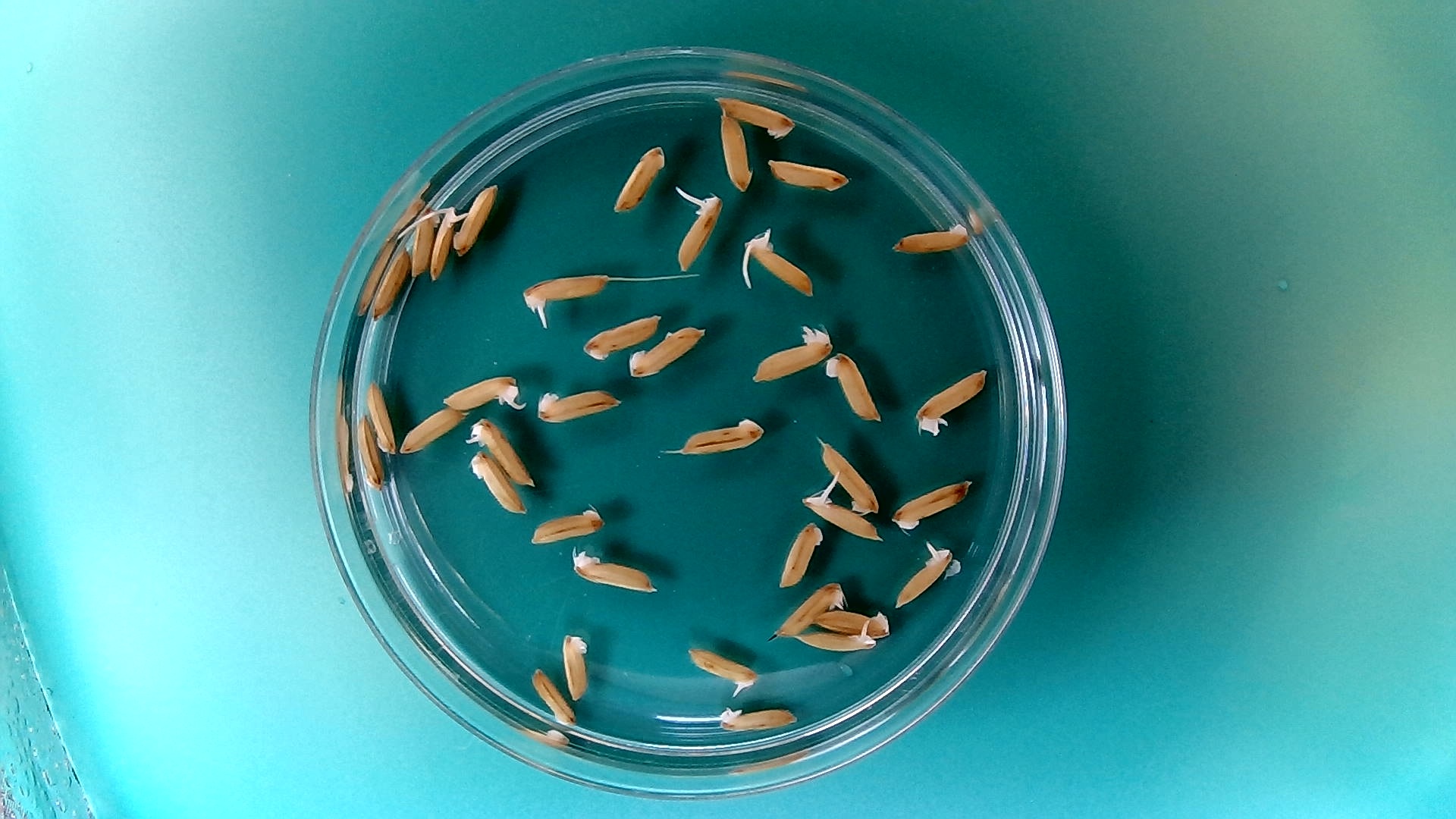


I3 I4


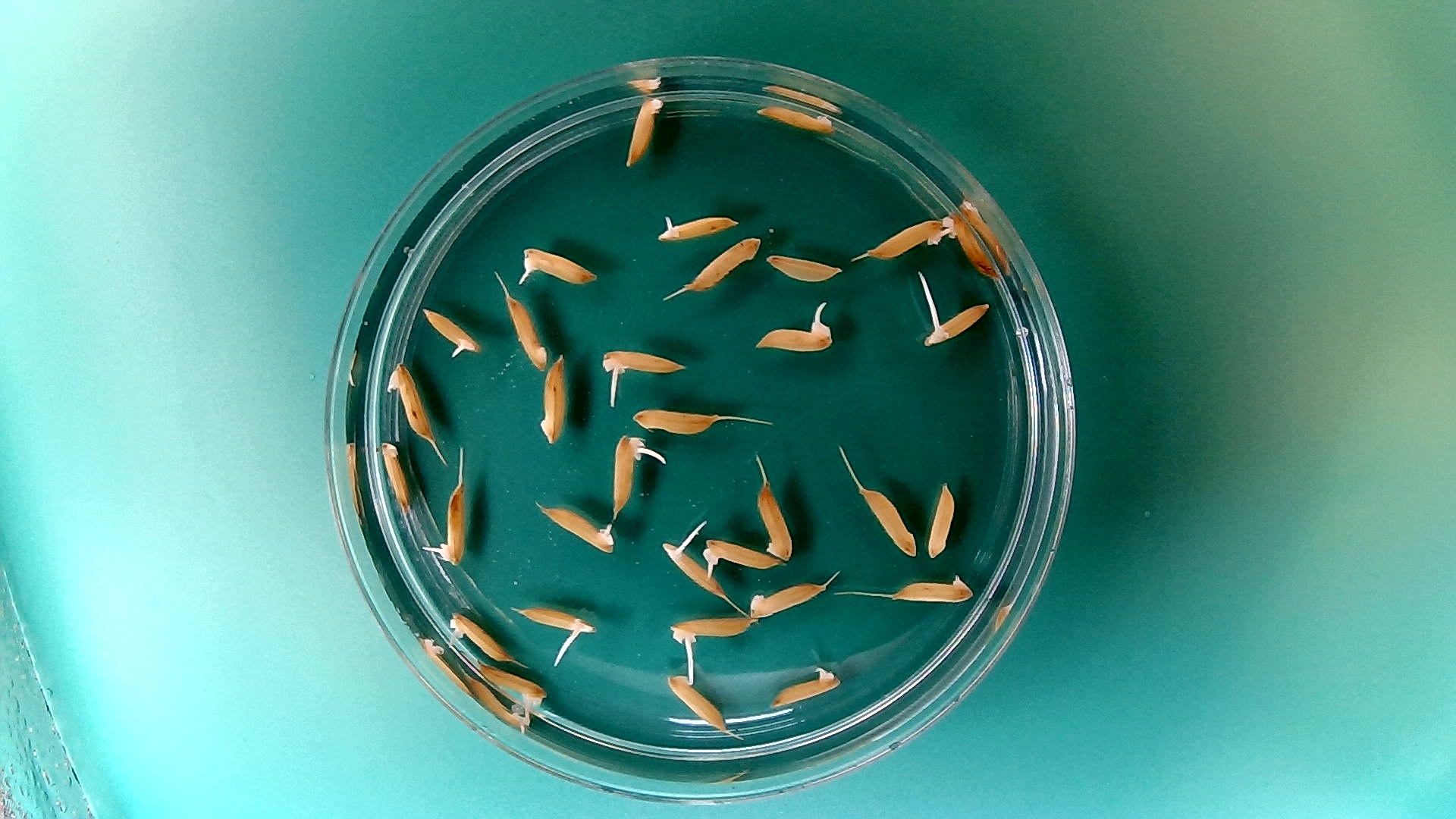


I5

Supplemental Figure S6 The five images involved in SeedRuler-SAM.


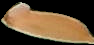

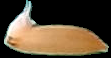

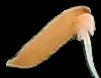


Criteria 1 Criteria 2 Criteria 3

Supplemental Figure S7 Three different germination standards.


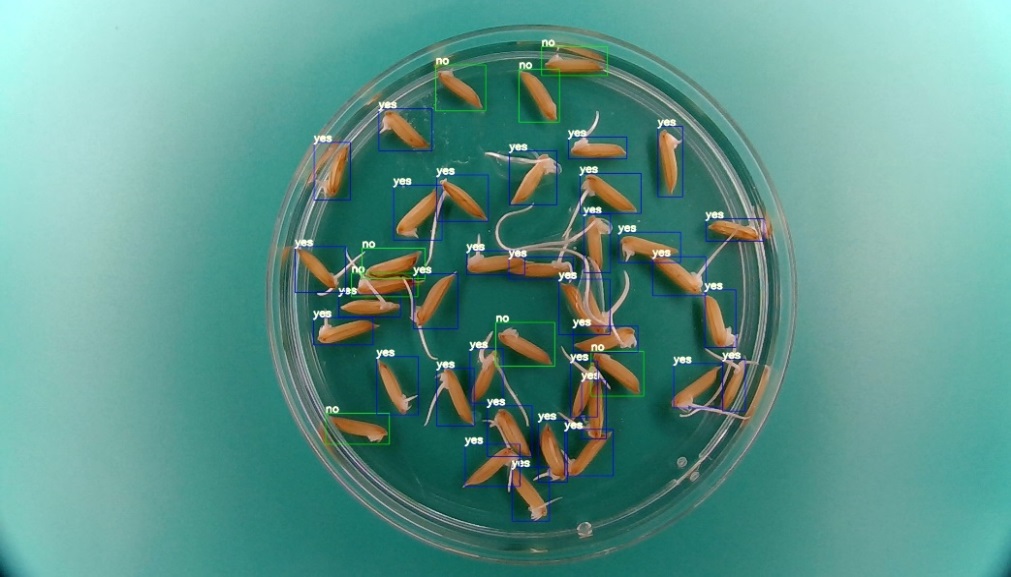

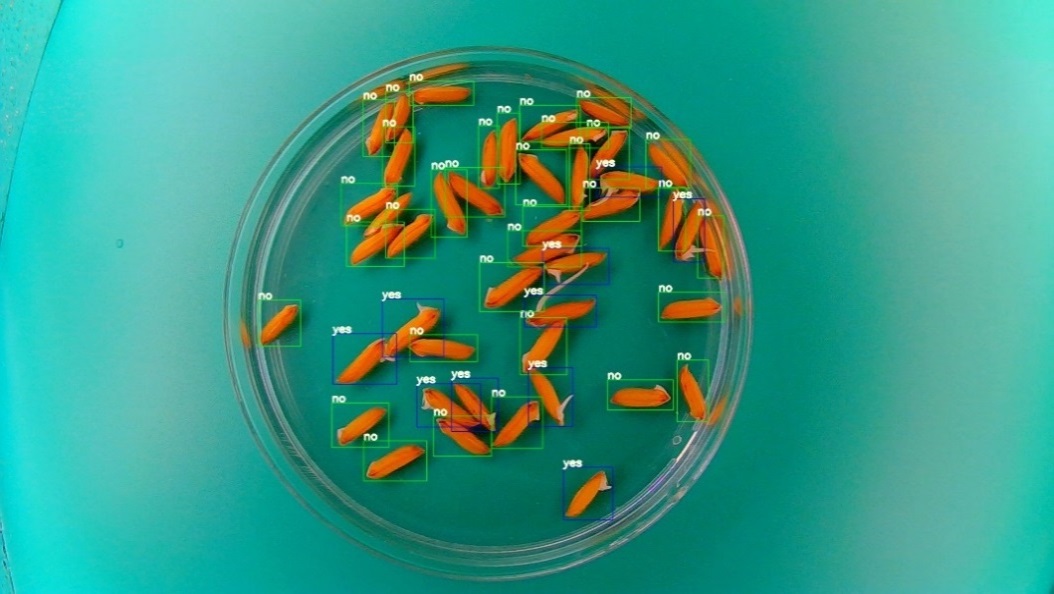


(a) (b)

Supplemental Figure S8 High overlap of the manually labeled boxes: (a) long radicles, (b) the seed distribution in image is denser.


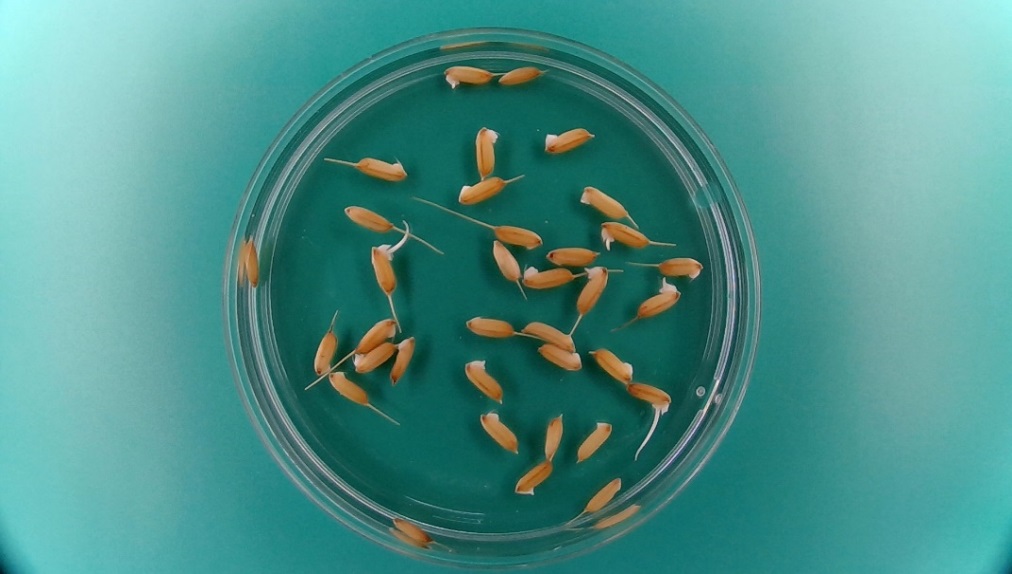

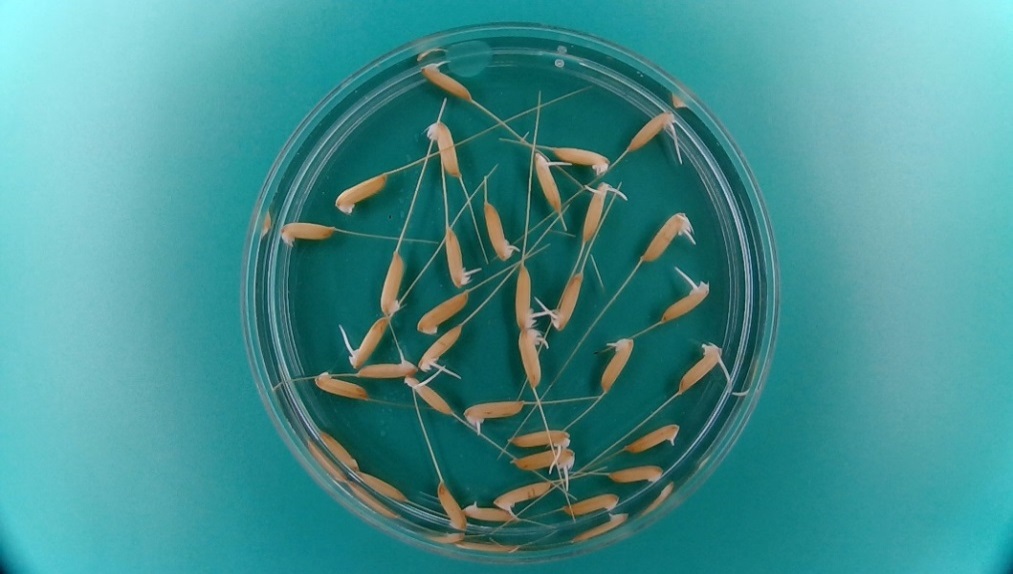


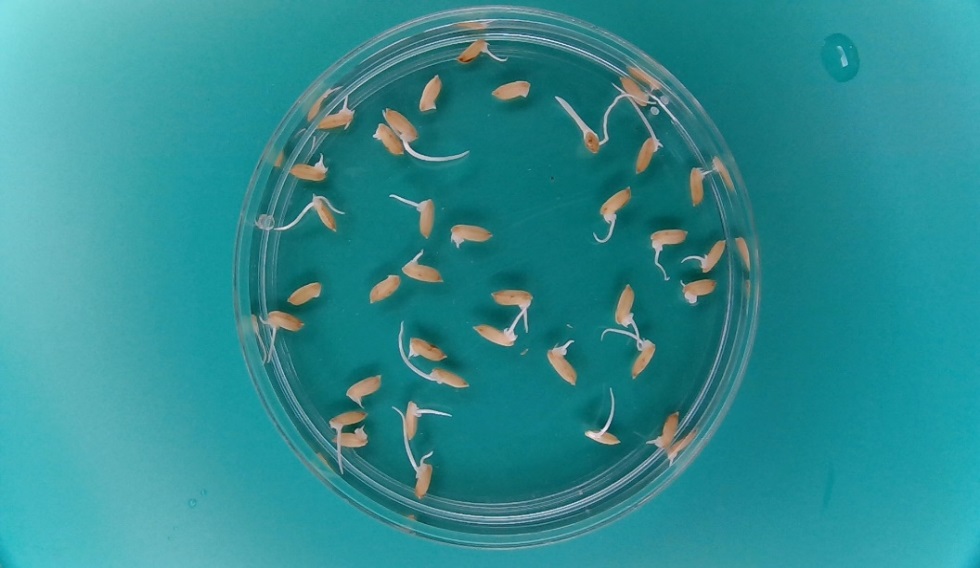

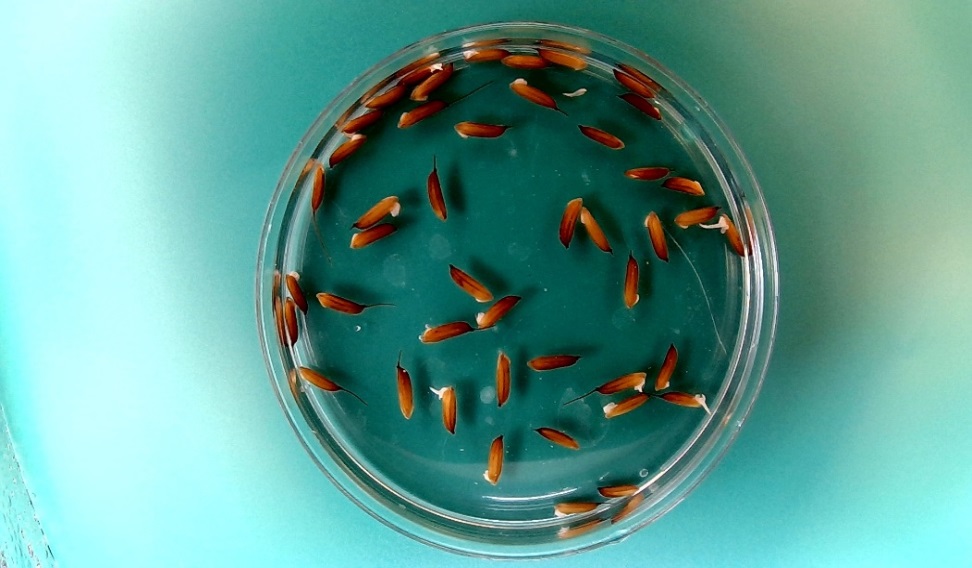


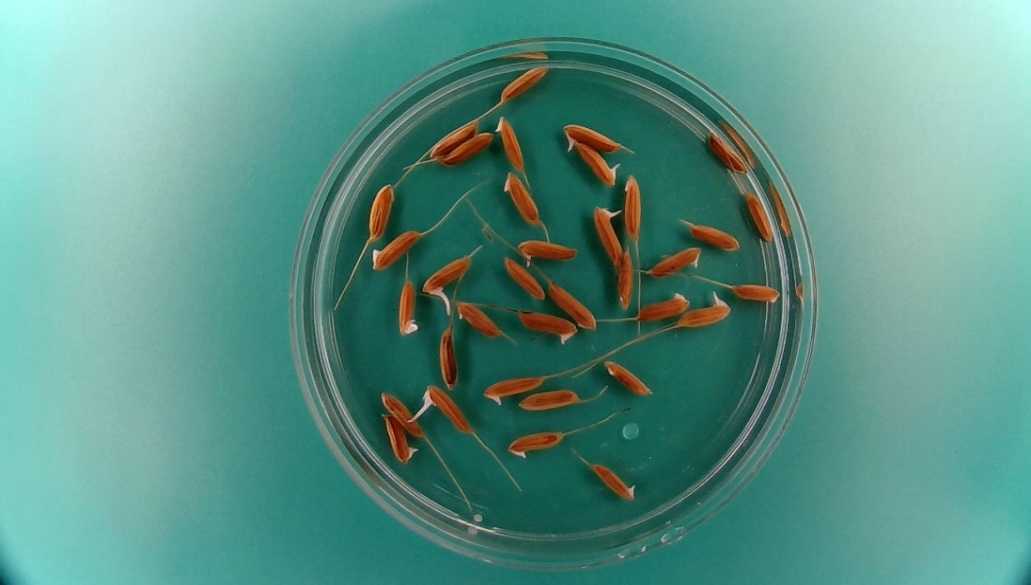

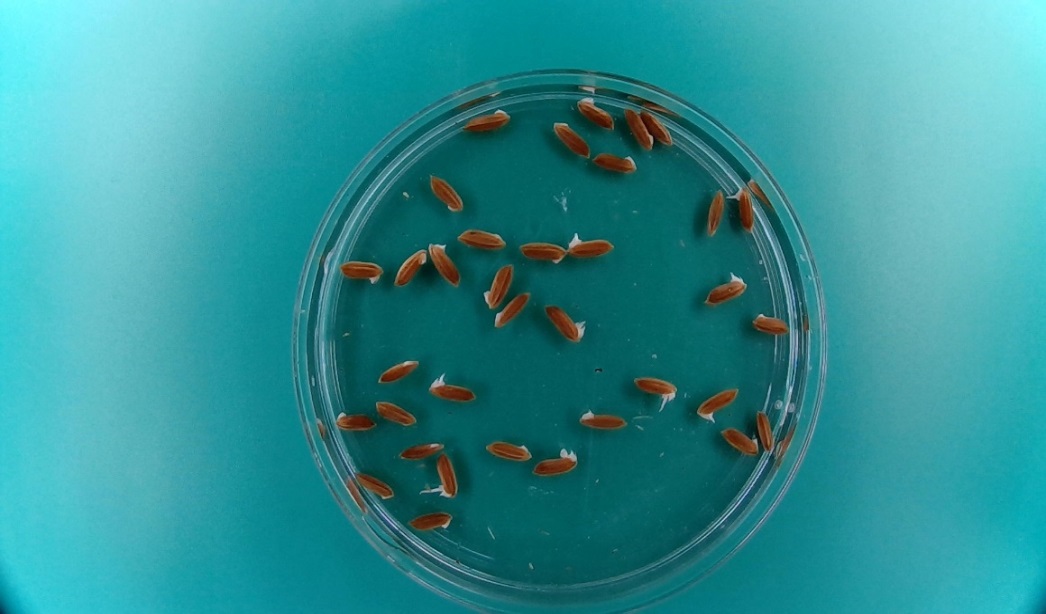


Supplemental Figure S9 A variety of rice seeds.


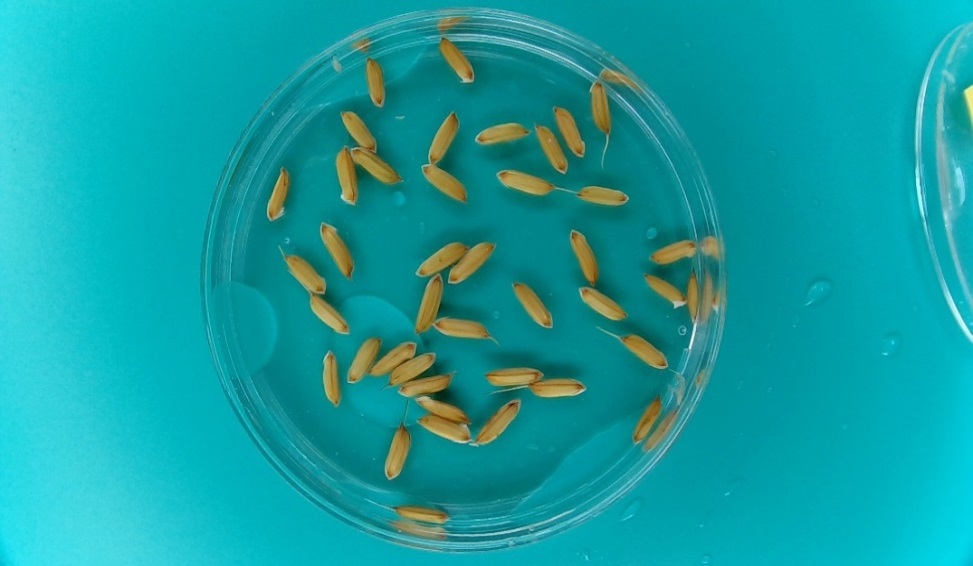

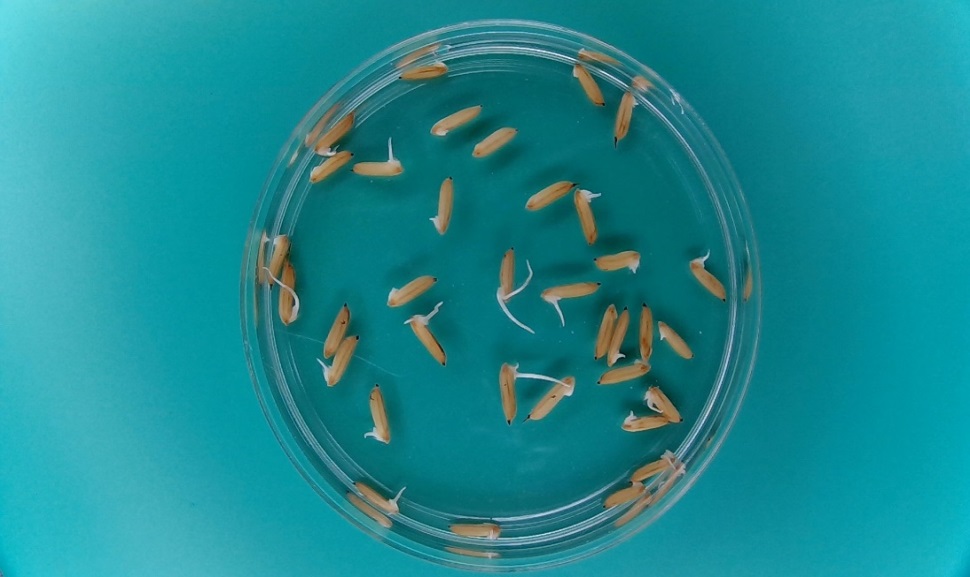


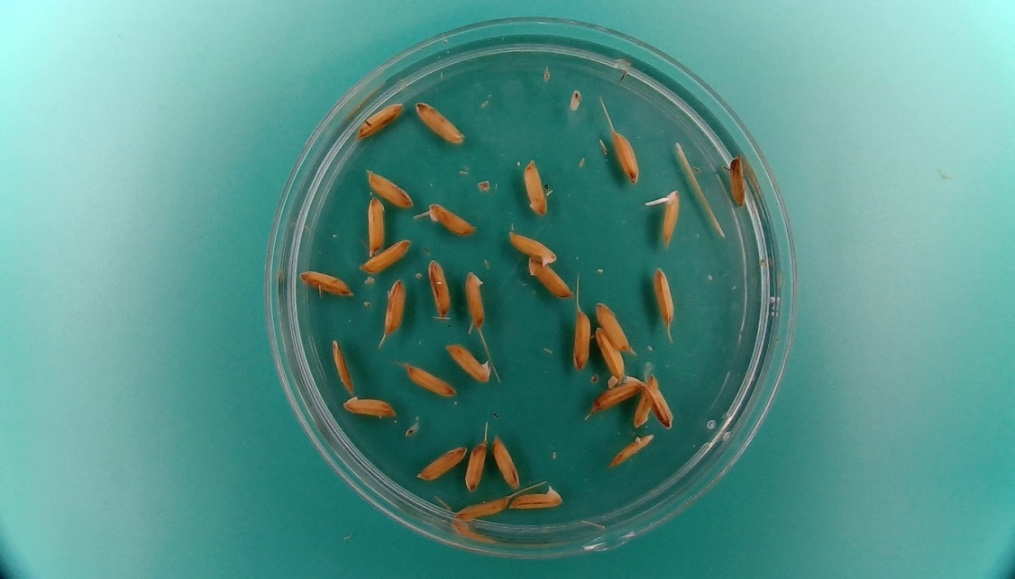

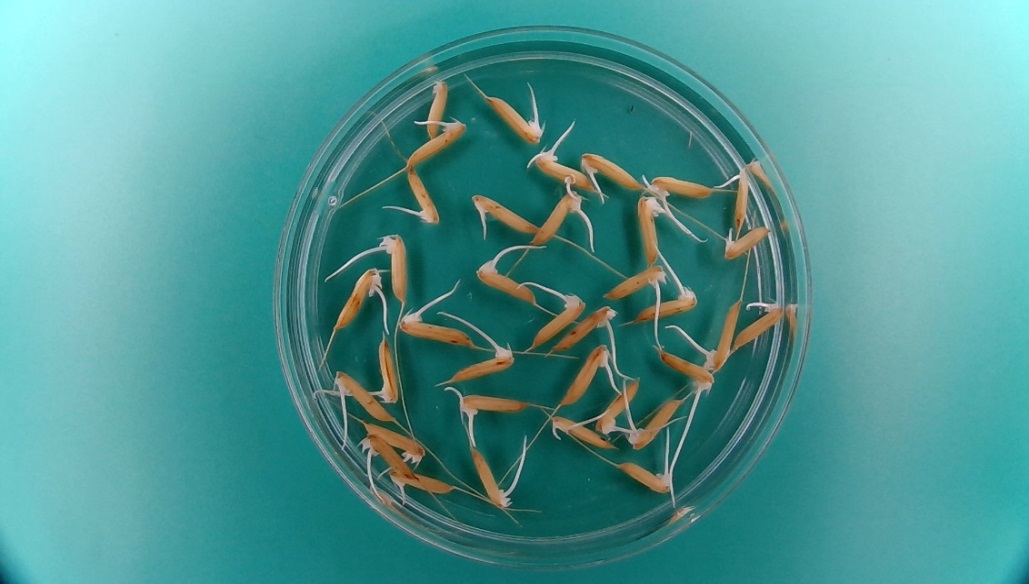


Supplemental Figure S10 The image contains water droplets, reflections, impurities, etc.


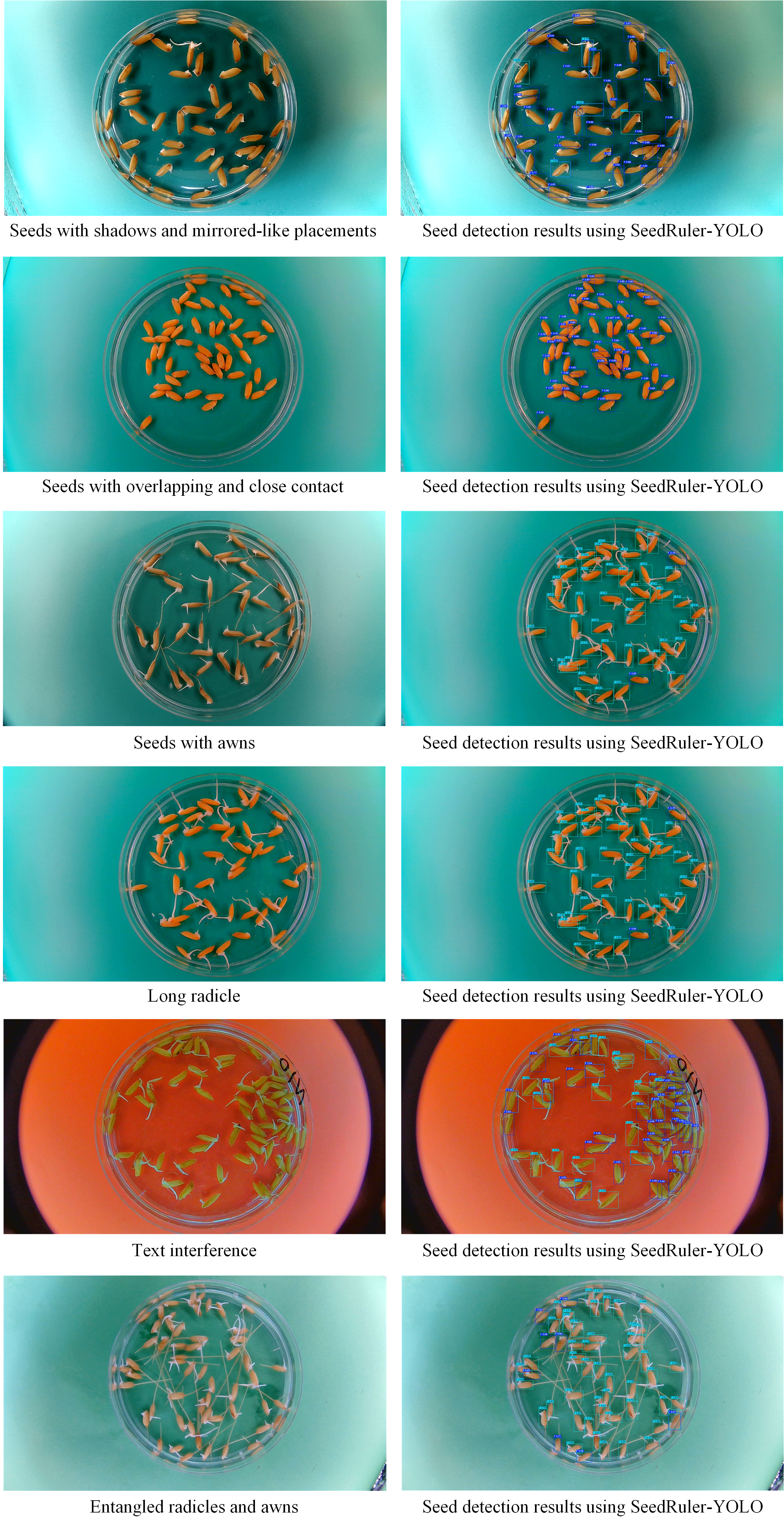


Supplemental Figure S11 Detection results of SeedRuler-YOLO under complex and challenging scenarios. Each pair of images shows the original input (left) and the corresponding detection results (right). Cyan boxes indicate germinated seeds, while blue boxes indicate non-germinated seeds. Scenarios include: (1) shadows and mirrored placements, (2) overlapping and close contact, (3) presence of awns, (4) long radicles, (5) background text interference, and (6) entangled radicles and awns.

Supplemental Table S1 The network structure of the four versions of YOLOv5, including YOLOv5s, YOLOv5m, YOLOv5l, and YOLOv5x

| Model | YOLOv5s | YOLOv5m | YOLOv5l | YOLOv5x |
| --- | --- | --- | --- | --- |
| Network layer number | 140 | 188 | 236 | 284 |
| Depth_multiple | 0.33 | 0.67 | 1 | 1.33 |
| Width_multiple | 0.50 | 0.75 | 1 | 1.25 |
| Number of BCSP | 1, 3 | 2, 6 | 3, 9 | 4, 12 |
| Convolution kernel number | 32, 64, 128, 256, 512 | 48, 96, 192, 384, 768 | 64, 128, 256, 512, 1024 | 80, 160, 320, 640, 1280 |
| Parameter number | 7.25e6 | 2.15e7 | 4.7e7 | 8.84e7 |

BCSP stands for Bottleneck Cross Stage Partial.

Depth_multiple controls the network depth, which refers to the number of BCSP layers. Width_multiple controls the network width, indicating the number of convolution kernels used.

Supplemental Table S2 Model performance obtained by taking images with a cell phone under natural lighting

| Metric | YOLOv5s | YOLOv5m | YOLOv5l | YOLOv5x |
| --- | --- | --- | --- | --- |
| mAP@0.5 | 0.871 | 0.929 | 0.925 | 0.916 |
| mAP@0.5:0.95 | 0.586 | 0.633 | 0.607 | 0.602 |
| MAE | 0.210 | 0.196 | 0.160 | 0.118 |
